# Comparative proteomic study of liver lipid droplets and mitochondria in mice housed at different temperatures

**DOI:** 10.1002/1873-3468.13509

**Published:** 2019-07-12

**Authors:** Qingfeng Liu, Ziyun Zhou, Pingsheng Liu, Shuyan Zhang

**Affiliations:** ^1^ School of Basic Medical Sciences Southwest Medical University Luzhou China; ^2^ National Laboratory of Biomacromolecules CAS Center for Excellence in Biomacromolecules Institute of Biophysics Chinese Academy of Sciences Beijing China; ^3^ University of Chinese Academy of Sciences Beijing China

**Keywords:** chronic cold stress, lipid droplet, liver adaptation, mitochondrion, organellar proteomics, thermoneutral temperature

## Abstract

Laboratory mice are standardly housed at around 23 °C, setting them under chronic cold stress. Metabolic changes in the liver in mice housed at thermoneutral, standard and cold temperatures remain unknown. In the present study, we isolated lipid droplets and mitochondria from their livers in a comparative proteomic study aiming to investigate the changes. According to proteomic analysis, mitochondrial tricarboxylic acid cycle (TCA cycle) and retinol metabolism are enhanced, whereas oxidative phosphorylation is not affected obviously under cold conditions, suggesting that liver mitochondria may increase TCA cycle capacity in biosynthetic pathways, as well as retinol metabolism, to help the liver to adapt. Based on proteomic and immunoblotting results, perilipin 5 and major urinary proteins are increased significantly, whereas mitochondrial pyruvate carrier is decreased dramatically under cold conditions, indicating their involvement in liver adaptation.

## Abbreviations

**ER**, endoplasmic reticulum

**H&E**, hematoxylin and eosin

**LC‐MS/MS,** liquid chromatography‐tandem mass spectroscopy

**LD**, lipid droplet

**MPC**, mitochondrial pyruvate carrier

**MT**, mitochondrion

**MUP**, major urinary protein

**PLIN5**, perilipin 5

**PNS**, post‐nuclear supernatant

**TAG**, triacylglycerol

**TCA cycle**, tricarboxylic acid cycle

**TEM**, transmission electron microscopy

**TM**, total membrane

**TMT**, tandem mass tag

***T***_**N**_, thermoneutral temperature

Currently the laboratory mouse, *Mus musculus*, serves as a powerful model system for studying metabolism and related disorders of human diseases 1, 2. For example, mouse genes can be easily manipulated to study their functions, and the relevant insights provided can be used to highlight the possible effect in humans. However, direct translation of any findings might be limited by differences of mouse and humans in their thermal physiology. The mammals apply a profound mechanism to conserve and utilize heat produced by metabolism to maintain a stable internal temperature in diverse environments. Thermoneutral temperature (*T*_N_) is a zone at which the metabolic rate (energy expenditure) required to maintain core body temperature is the lowest 3. For most used laboratory mouse, C57BL/6J, *T*_N_ is around 29–31 °C 2, 4, 5. For naked human, *T*_N_ is around 28 °C 6, 7, 8. For clothed humans (i.e. clothed animal handlers), *T*_N_ is about 20–22 °C 9. Thus, for the comfort of handlers, laboratory mice are housed at standard temperature of around 20–23 °C 1. Consequently, standardly housed mice undergo thermal stress constantly, resulting in a dramatic alteration of their physiology and immune responses 10. Furthermore, mice housed at a standard temperature fail to mimic a number of human diseases 11, 12, 13, 14. Ganeshan *et al*. 9 commented that warming the laboratory mouse might allow for more predictive modelling of human diseases and therapies.

The liver plays a key role in the modulation of whole‐body energy balance and fuel availability. Dysfunction of liver metabolism can cause a series of metabolic diseases, such as nonalcoholic fatty liver disease, type 2 diabetes and cardiovascular diseases. From a metabolic perspective, the liver is expected to undergo profound metabolic changes under chronic cold conditions to meet the metabolic demands. In 2017, for the first time, Simcox *et al*. 15 identified the liver as an essential site for cold adaptation and found that it provides acylcarnitines as fuel for peripheral tissues, including brown adipose tissue (BAT), heart and skeletal muscle during cold exposure.

However, the exact physiological changes occurring in the liver in the laboratory mouse under chronic cold stress are unclear. Energy reservoirs in liver are composed of glycogen and triacylglycerol (TAG). TAG is stored in lipid droplets (LDs). LD comprises a metabolic active organelle, with a mono‐phospholipid membrane surrounding a neutral lipids core, involved in multiple cellular processes 16, 17. For example, LDs are an active site for lipid metabolism 18. Ectopic storage of lipids in LDs is linked to human metabolic syndrome 19. The mitochondrion (MT) is another major organelle regulating metabolism. Mitochondria host the machinery for oxidative phosphorylation, the most efficient pathway to supply ATP for cell. Furthermore, mitochondria include all the proteins for the tricarboxylic acid cycle (TCA cycle), which plays a central role in the break down organic fuel molecules, such as glucose, fatty acids and amino acids. The TCA cycle is also important in the biosynthetic processes in which the intermediates leave the cycle to be synthesized as glucose, fatty acids or amino acids 20, 21.

To understand how the mouse liver adapts to housing conditions, a bottom‐up strategy, conducting organellar proteomics other than whole tissue proteomics, was performed. We compared LDs and mitochondria from liver in laboratory mouse living in their *T*_N_ *T*_30_, commonly housed temperature *T*_23_, as well as extremely cold temperature *T*_6_, respectively. The results showed that, under 4‐week cold acclimation, glycogen in the liver was decreased compared to under *T*_N_. Comparative proteomic results showed an increase in mitochondrial TCA cycle and retinol metabolism in liver of mouse under extreme cold temperature but no change in oxidative phosphorylation. We found that mitochondrial pyruvate carrier (MPC), major urinary protein (MUP) and perilipin 5 (PLIN5) may play roles in the regulation of liver metabolism.

## Materials and methods

### Materials

The Triglyceride Kit (GPO‐PAP Method) was purchased from BioSino Bio‐Technology & Science Incorporated (Beijing, China). The EnzyChrom™ Glycogen Assay Kit was obtained from BioAssay Systems (Hayward, CA, USA). Twenty‐five percent glutaraldehyde solution, 8% paraformaldehyde solution, EMbed 812 kit, uranyl acetate and lead citrate were all obtained from Electron Microscopy Sciences (Hatfield, PA, USA). Osmium tetraoxide (EM grade) was obtained from Nakalai Tesque (Kytoto, Japan). Potassium ferrocyanide was obtained from Sigma‐Aldrich (St Louis, MO, USA). Pierce™ BCA Protein Assay Kit, HCS LipidTOX™ Red Neutral Lipid Stain and MitoTracker™ Red CMXRos were obtained from Thermo Fisher Scientific (Waltham, MA, USA).

### Animals

All animal experiments were approved by the Committee of Biosafety, Ethics and Experimental Animal Management of Institute of Biophysics, Chinese Academy of Sciences, permit number SYXK (Jing) 2016‐0026. All the procedures were performed in accordance with the NIH Guide for the Care and Use of Laboratory Animals (8th edition). Ten‐week‐old male C57BL/6 mice were obtained from Beijing Vital River Laboratories (Beijing, China). They were randomly separated into three groups and housed at *T*_N_ (30 °C, *T*_30_), standard temperature (23 °C, *T*_23_) and cold temperature (6 °C, *T*_6_), respectively. The mice were all maintained in individual cages under 12:12 h light/dark cycles and fed standard laboratory chow for 4 weeks.

### Histological and ultrastructural analysis of mouse liver

After being housed for 4 weeks at three temperatures, liver histology was analyzed by hematoxylin and eosin (H&E) staining. Briefly, the samples of liver were collected carefully and fixed immediately in 4% (w/v) paraformaldehyde for 24 h and washed with 70% ethanol solution. After dehydration in ethanol series, the samples were embedded in paraffin. The paraffin blocks were sectioned and then stained with H&E dyes for observation.

The ultra‐structure of the liver was analyzed by transmission electron microscopy (TEM). Samples of mouse liver were quickly removed and fixed immediately in 2.5% (w/v) glutaraldehyde in 0.1 m phosphate buffer (pH 7.2) and then cut into small pieces (approximately 1 mm^3^). Then, the samples were fixed at 4 °C overnight and afterwards fixed in 1% (w/v) osmium tetraoxide (with 0.8% potassium ferrocyanide) for 2.5 h at room temperature. After staining in 2% (w/v) uranyl acetate overnight at 4 °C, the samples were dehydrated in an ascending concentration series of ethanol at room temperature. Then they were embedded in EMbed 812 and prepared as 70 nm sections. After staining with uranyl acetate and subsequently with lead citrate, the sections were viewed with Tecnai Spirit (Thermo Fisher Scientific) electron microscope.

### Measurement of liver TAG content

The content of liver TAG was measured using a Triglycerides Kit (GPO‐PAP Method) in accordance with the manufacturer's instructions. Approximately 20 mg of liver tissue was washed in cold PBS, and then suspended in 1% Triton X‐100 and subsequently homogenized using a Dounce homogenizer. The homogenate was centrifuged and subsequently TAG in the sample was hydrolyzed and released glycerol was analyzed enzymatically. *A*_505_ of the resulting product was read in a spectrophotometer and then the TAG content was calculated.

### Measurement of liver glycogen content

The liver glycogen content was determined by using the EnzyChrom™ Glycogen Assay Kit in accordance with manufacturer's instructions. Approximately 10 mg of liver tissue was washed in cold PBS, and then homogenized with a Dounce homogenizer. The homogenates were boiled for 10 min and centrifuged at 18 000 ***g*** for 10 min at 4 °C. The supernatant was transferred into a new tube for use.

The resulting supernatant was then mixed with the working reagent in the kit for 30 min to break down glycogen and oxidize glucose and finally produce a product generating color for detection. *A*_570_ was read in a spectrophotometer. The glycogen content was then calculated by comparing with the standard reading values.

### Isolation of LDs from liver

LDs were isolated from liver based on our previous method with some modifications 22. Briefly, liver was carefully collected and rinsed in cold saline. Subsequently, the liver was transferred into 10 mL of Buffer A (25 mm tricine, pH 7.8, 250 mm sucrose) containing 0.2 mm PMSF and sliced into small pieces using tweezers. After being homogenized with a loose‐fitting glass‐Teflon Dounce, the homogenate was centrifuged at 1000 ***g*** for 10 min and the supernatant was the post‐nuclear supernatant (PNS). Then PNS was transferred into a SW40 Ti tube and Buffer B (20 mm Hepes, pH 7.4, 100 mm KCl and 2 mm MgCl_2_) was overlaid on the top. The gradient was centrifuged at 13 000 ***g*** for 1 h at 4 °C. The LD layer on the top was collected carefully and washed for use.

### Isolation of mitochondria from liver

Mitochondria were isolated according to our previous method with some modifications 23. The above PNS was centrifuged at 8000 ***g*** for 10 min at 4 °C. Then, the supernatant was discarded and the pellet was resuspended with Buffer B and washed three times. Then, the mitochondrial suspension was gently loaded on the top of a Percoll step gradient consisting of an upper 25% Percoll (8 mL) and a lower 50% Percoll (3 mL) layer. Subsequently, the gradient was centrifuged at 41 000 ***g*** for 1 h at 4 °C and then the mitochondria were recovered from the 50% to 25% Percoll interface and washed with Buffer B for use.

### Confocal microscopy analysis of isolated LDs and mitochondria

The isolated LDs and mitochondria were stained on ice for 30 min using LipidTOX Red (dilution 1:500, v/v) (Thermo Fisher Scientific) and MitoTracker Red (dilution 1:500, v/v) (Waltham, MA, USA) respectively. Next, they were mounted onto glass slides and cover slips for visualization using a FV1200 Imaging System (Olympus, Tokyo, Japan).

### Preparation and analysis of proteins and lipids

After collection, LDs and mitochondria were treated with acetone‐chloroform (5:1, v/v) and strongly vortexed, respectively. The tubes were centrifuged at 20 000 ***g*** for 10 min and proteins were pelleted. The proteins were then analyzed by silver staining or western blotting.

For LDs, the supernatant lipid fraction was transferred to a new tube and evaporated under a stream of nitrogen. Lipid was developed by TLC using solvent of hexane‐diethyl ether‐acetic acid (80 : 20 : 1, v/v/v). The TLC plate was stained by iodine vapor at room temperature.

### Mass spectrometry and data analysis

For comparative proteomic study, two groups of mice (each with three mice), at each temperature were used. The liver LD or mitochondrial protein samples from those six groups were labelled by tandem mass tag (TMT) 6plex kit (Thermo Fisher Scientific) following the TMT 6plex Reagents Protocols. Detailed procedures are provided in Appendix S1. Briefly, the precipitated protein was reduced and digested with trypsin. For mitochondrial samples, the digested peptides were desalted. The peptides were labelled by TMT 6plex in accordance with the manufacturer's instructions. Then, the labelled peptides were separated by nano liquid chromatography‐tandem mass spectroscopy (LC‐MS/MS) using a Q Exactive equipped with an Easy‐nLC 1000 HPLC system (Thermo Fisher Scientific). The raw data were extracted and quantitated using thermo proteome discoverer 2.2.0.388 (Thermo Fisher Scientific). The KEGG database was used for gene functional categorization and pathway analysis. The STRING database was adopted to map the interaction (https://string-db.org). The DAVID database (https://david.ncifcrf.gov/home.jsp) was used to analyze proteins in KEGG pathways.

## Results

### Effects of thermoneutral temperature and chronic cold exposure on mouse liver

Because the model animals are usually housed at a standard temperature of around 22 °C, which is lower than their *T*_N_ 30 °C, and the liver is a key metabolic organ, we wanted to determine how environmental temperature influences the metabolism in the mouse liver. Hence, laboratory mice C57BL/6, approximately 10 weeks old, were randomly allocated into three groups and then housed one per cage at temperatures of 30, 23 and 6 °C independently for 4 weeks. For each temperature, six mice were randomly separated into two groups (i.e. three for each).

**Figure 1:**
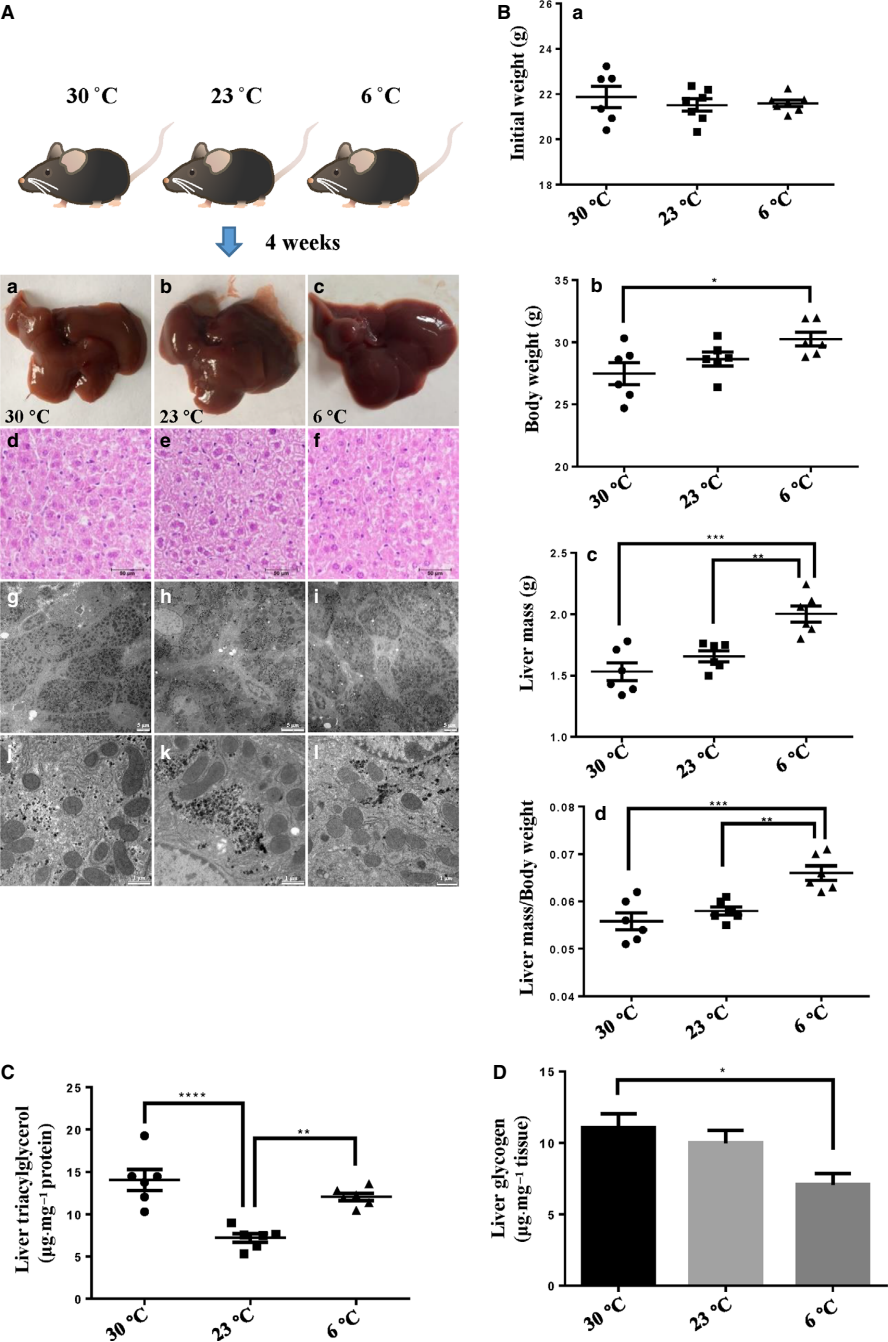
The changes of male mouse livers when mice were housed at *T*_N_, standard temperature and cold temperatures for 4 weeks. The male mice aged 10 weeks were housed in individual cages at 30, 23 or 6 °C for 4 weeks and fed a chow diet. (A) Histological and ultrastructural analysis of mouse livers after housing for 4 weeks. (Aa–Ac) Liver morphology. (Ad–Af) Liver histology by H&E staining of liver sections. (Ag–Al) Liver ultrastructure analyzed by TEM. (B) Effects of housing temperature on liver mass and body weight after housing for 4 weeks. (Ba) Initial body weight. (Bb) Final body weight. (Bc) Liver mass. (Bd) Ratio of liver mass to body weight. (C) TAG content in liver after housing for 4 weeks. (D) Glycogen content in liver after housing for 4 weeks. In (B) to (D), *n *=* *6 per group. In the bar graphs, the data represent the mean ± SEM, analyzed by one‐way ANOVA. *P <* *0.05; **P <* *0.01; ***P <* *0.001; ****P <* *0.0001.

Under three temperatures, all animals had been well. Compared to the morphology of the liver from standardly housed mice, which is the current well‐known liver morphology, the livers from mice living in thermoneutral and extreme cold temperatures exhibited no anatomic changes, although their sizes differed (i.e. the colder, the bigger) (Fig. 1A). The livers under three temperatures showed no obvious differences in histological morphology as indicated by H&E staining. Ultra‐thin sections of livers revealed that, when the environmental temperature declined, LDs became smaller and mitochondria showed no apparent changes. The body weights of mice housed at 6 °C were higher than those of the other two groups after 4 weeks of acclimation (Fig. 1B). The absolute liver masses differed among the three groups, with a tendency for heavier livers in colder environments. The relative liver mass (liver mass/whole body weight) in extreme cold temperatures was significantly increased compared to that at *T*_30_ and *T*_23_. These results are consistent with previous studies 10, 24, 25.

Because the liver is an essential organ with respect to providing fuel, we further tested the energy reservoir, glycogen and TAG content under the three temperatures. TAG contents under thermoneutral and extreme cold conditions were higher than that in the standard condition (Fig. 1C). Glycogen under extreme conditions was lower than that under thermoneutral conditions (Fig. 1D). All of these results indicate that the chronic cold temperature to which the laboratory mouse is always exposed can result in remarkable changes in the liver with respect to adapting to the whole‐body demand. To determine the exact critical metabolic alteration, we carried out a comparative proteomic study on the key metabolic organelles, LDs and mitochondria involved in the changes and investigated how they help liver to adapt to distinct environments.

### Isolation and quality verification of lipid droplets and mitochondria from livers at three temperatures

**Figure 2:**
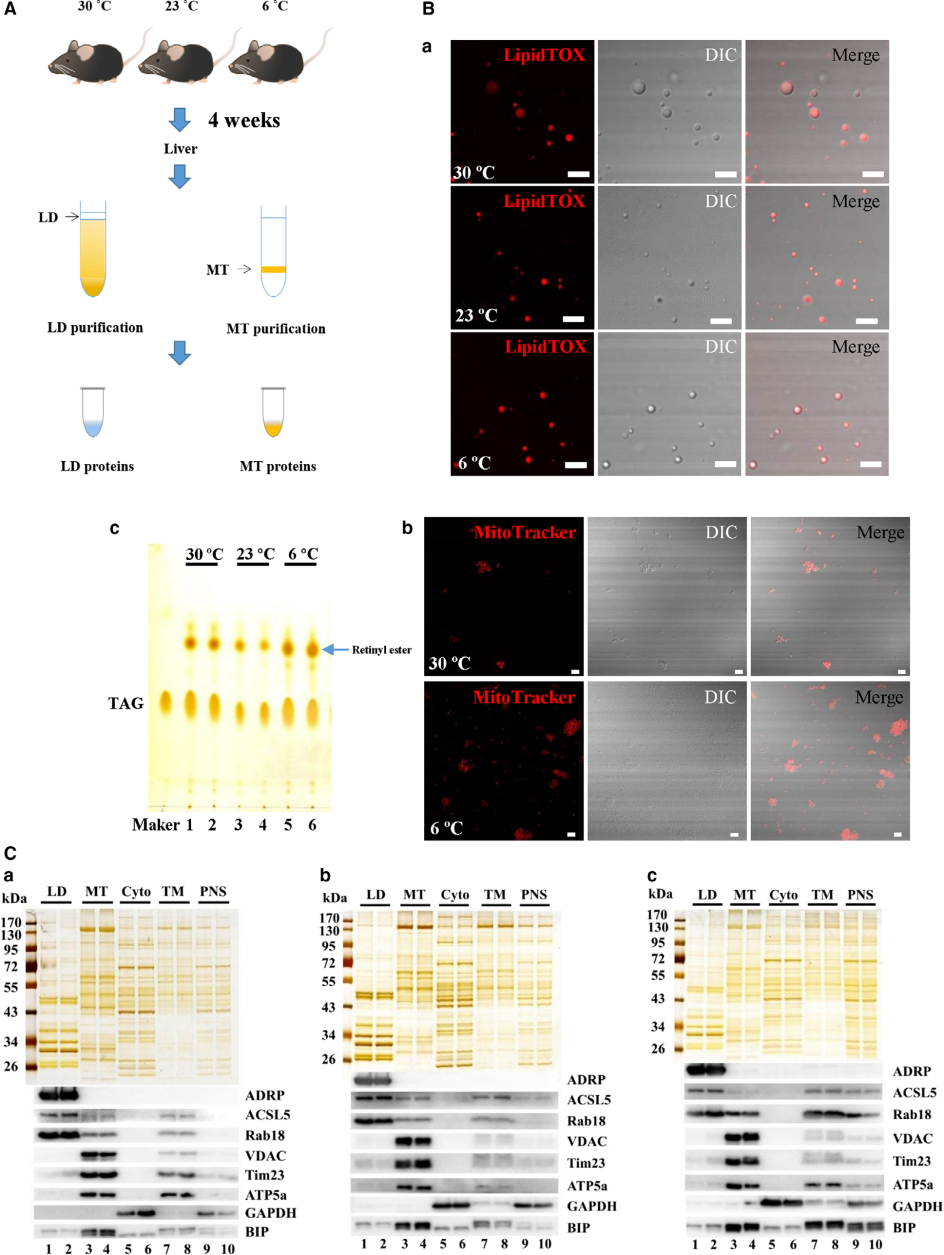
Verification of purified LDs and mitochondria. LDs and mitochondria were isolated from the livers of mice after being housed at different temperatures for 4 weeks. (A) Flowchart for isolation. Briefly, LDs were isolated using sucrose density centrifugation and the resulting top layer was collected. Mitochondria were isolated using Percoll density gradient centrifugation and the resulting layer from the 50% to 25% Percoll interface was recovered. (Ba) Confocal microscopy analysis of isolated LDs by LipidTOX Red staining, DIC imaging and merged images. (Bb) Confocal microscopy analysis of isolated mitochondria by MitoTracker Red staining, DIC imaging and merged images. (Bc) Analysis of lipids extracted from isolated LDs by TLC. Scale Bar = 5 μm. (C) Silver staining and western blotting analysis of fractions from livers in mice housed at (Ca) 30 °C (Cb) 23 °C and (Cc) 6 °C. The proteins from isolated LD, MT, cytosol (Cyto), TM and PNS fractions were separated by SDS/PAGE and silver stained (upper). With equal protein loading, the indicated antibodies were tested to probe for marker proteins of different organelles/cellular fractions (lower): ADRP, ACSL5 and Rab18 (LD proteins); VDAC, Tim23 and ATP5a (MT proteins); GAPDH (cytosol protein); and BIP (ER protein).

To study organelle proteomics, first we isolated the LDs and mitochondria from livers in mice living under three temperatures. LDs were isolated in accordance with previously established protocols (Fig. 2A) 22. The quality of the isolated LDs was verified by LipidTOX Red staining and differential interference contrast (DIC) imaging analysis (Fig. 2Ba). The image showed the LipidTOX staining LDs and DIC imaging spherical structure were well overlapped, indicating a high‐quality preparation of LDs. TLC analysis of lipids of isolated LDs showed a low phospholipid ratio, also suggesting no obvious membrane contamination (Fig. 2Bc). Also, the TLC result was in agreement with our above results regarding the liver TAG change pattern under three temperatures. Notably, the spot, validated to be retinyl ester 26, increased in the liver LDs from mice living under a cold temperature (Fig. 2Bc). LD is the main storage site of retinyl esters in liver. Therefore, on exposure to cold, retinyl ester may play a role in helping the liver to adapt.

The quality of isolated LDs was also verified by biochemical methods. As indicated by silver staining (Fig. 2C, upper), the protein pattern of isolated LDs was unique compared to the other three cellular fractions, cytosol, total membrane (TM) and PNS, indicating a great enrichment of LD‐associated proteins. Two independent LD preparations were analyzed and they exhibited almost identical protein profiles, indicating a good reproducibility of LD preparation. The purity of LD fraction was further assessed by immunoblotting with the indicated antibodies (Fig. 2C, lower). Equal amounts of proteins from LD, cytosol, TM and PNS were blotted. The results show that PLIN2/ADRP, a marker protein of mammal LDs, was detected only in the LD fraction. ACSL5 and Rab18, the other two LD‐associated proteins, were also enriched in the LD fraction. Marker proteins for mitochondria (VDAC, Tim23 and ATP5a), cytosol (GAPDH) and endoplasmic reticulum (ER) (BIP) were barely detected in the LD fraction. These results confirmed a high purity of isolated LDs.

Similarly, the purity of isolated mitochondria was analyzed. The results obtained showed that the MtioTracker Red staining and DIC imaging were merged well (Fig. 2Bb). Protein profile of the isolated mitochondria was significantly different from the other fractions (Fig. 2C, upper). A good reproducibility was confirmed by the identical protein profiles between two independent purifications. Immunoblotting analysis showed great enrichment of mitochondrial maker proteins in the isolated mitochondrial fraction, whereas other cellular proteins were barely detected (Fig. 2C, lower). The ER marker protein was also found in the mitochondrial fraction, indicating a tight association between ER and mitochondria, which is in agreement with the MAM structure 27. These data confirmed a high purity of the isolated mitochondrial fraction.

### Proteomic profiling of mouse liver LDs and mitochondria

**Figure 3:**
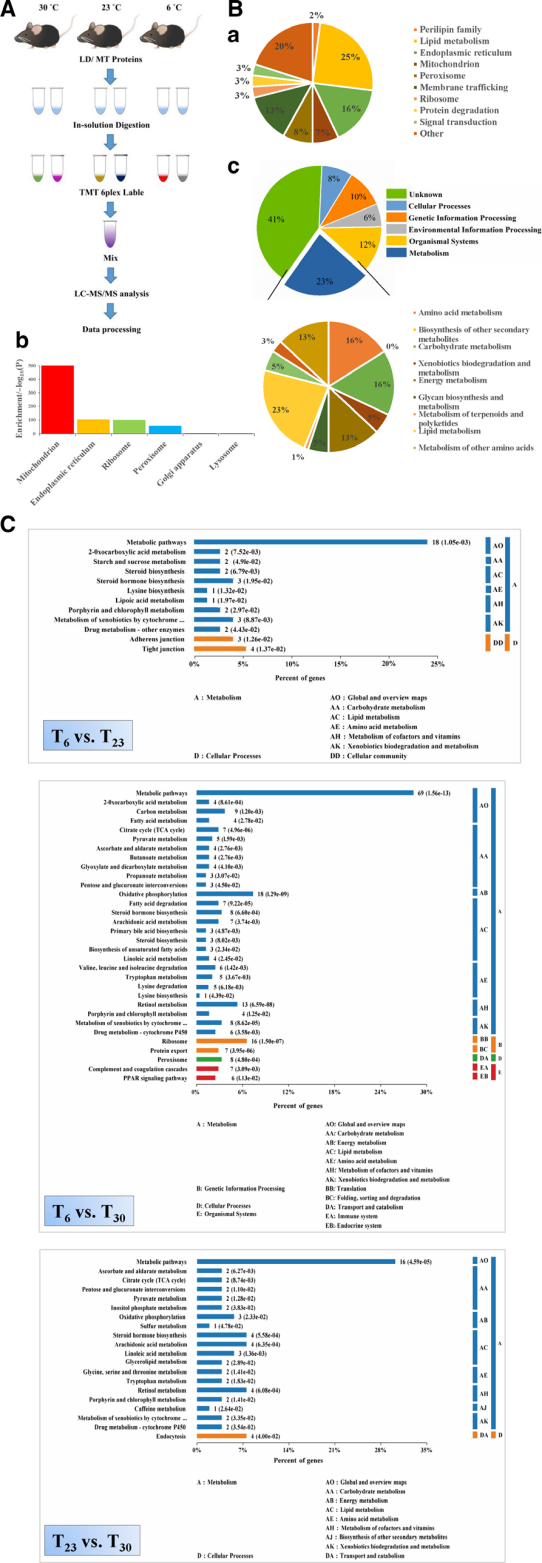
TMT‐based comparative proteomic analysis of liver LDs and mitochondria from mouse living at different temperatures. The LD and mitochondrial proteins were labelled with TMT for comparative proteomic study, respectively. (A) Flow chart of the experimental procedures. Briefly, the LD or mitochondrial proteins from six groups (two groups in each temperature) were digested. After labelling with TMT 6plex reagent and separation, thermo proteome discoverer 2.2.0.388 was used to search the raw data for protein identification and quantitation. (B) Overview of the identified proteins. (Ba) The identified LD proteins were analyzed and categorized by subcellular locations and functions according to the UniProtKB database. (Bb) The identified proteins in the mitochondrial preparations were subjected to enrichment analysis. The DAVID Bioinformatics tool was used and the enrichment factors were calculated for cellular organelles. (Bc) The identified mitochondrial proteins were categorized according to the KEGG pathway system. (C) Pathway enrichment analysis of the differential proteins in liver mitochondria under different conditions. The differential proteins were subjected to a KEGG pathway enrichment analysis. The ratio of the enriched differential proteins in a specific pathway to the total differential proteins was shown. The protein amount and the *P* value are also shown. *P* represents the significance of each pathway enriched by the mitochondrial proteins. *P* < 0.05 is considered statistically significant with respect to consideration.

After isolated LDs and mitochondria were shown to be high quality, a comparative proteomic study was carried out to gain insights into how the proteins of these two important organelles were altered under three housing temperatures. Figure 3A shows the schematic workflow. Briefly, LD proteins and mitochondrial proteins from two independent groups under each temperature were digested with trypsin. Then, the peptides were labelled with the indicated isobaric TMT. After mixing, the labelled peptides were subjected to nano LC‐MS/MS analysis. The proteins were identified using thermo proteome discoverer 2.2.0.388 and those with at least two peptides with 99% confidence (false discovery rate of 1%) were selected for further analysis.

In the LD proteome, 184 proteins were identified and categorized into 10 groups based on their cellular functions and subcellular locations according to the UniProt database (Fig. 3Ba and Table S1). Among the identified proteins, 114 (62% of the total) have been reported in previous LD proteomes or confirmed on LDs by imaging and the ratio is similar to the published LD proteomes 28, 29, which indicates the reliability of the isolation and proteomic techniques (Table S1). Three perilipin family proteins, PLIN2, PLIN5 and PLIN1, were identified. The most abundant proteins identified were involved in lipid metabolism (25%; 46 proteins), which is consistent with previous reports on liver LD proteomics 30. In addition, in our previous study, we found that isolated LDs, almost depleted of ER, were able to incorporate radiolabelled fatty acids into TAG and phospholipids 18. Recently, we found adrenal LD may be important sites for steroid hormone metabolism 31. All of these results confirmed the role of LD as a lipid metabolic organelle for maintaining the cellular lipid homeostasis 32. Furthermore, ER, mitochondrial and peroxisome proteins constituted 16% (29 proteins), 7% (13 proteins) and 8% (14 proteins) of the total proteins, respectively. Proteins of those organelles were also observed in other LD proteomic studies, which suggested that LD dynamically interacts with other intracellular organelles 17, 33. Another major group consisted of 24 proteins involved in membrane trafficking (approximately 13% of the total) that exhibit important roles in LD dynamics and interactions with other cellular organelles 34, 35. Approximately 3% of the identified proteins (five proteins) were ribosome proteins, which have also been reported in other proteomic studies 36, 37. Another 3% of the proteome (five proteins) comprised proteins involved in protein degradation. LDs were found to play a role in the degradation of apolipoprotein B‐100 and HMG‐CoA reductase and proposed to be functionally involved in protein degradation 38, 39, 40. Six proteins involved in the signalling pathway comprised 3% of the total proteins, supporting the hypothesis that LDs are involved in signal transduction 41. Moreover, a large number of other proteins (20%; 39 proteins) were identified, indicating the additional biological roles that LD may play.

In the mitochondrial proteome, 1886 proteins were identified in total (Table S2). To determine the reliability of our mitochondrial proteome, enrichment of the identified proteins in cellular component was analyzed using the DAVID Bioinformatics tool (https://david.ncifcrf.gov/home.jsp). It was clearly shown that mitochondrial proteins were greatly enriched (Fig. 3Bb). In addition, the identified proteins were analyzed using the DAVID database and compared with the mitochondrial database mitocarta2.0 42. Nine hundred and four of the 1886 total identified proteins (48% of the total) were annotated as mitochondrial proteins. This ratio is similar to that reported in the previous study on the mitochondrial proteome of mouse liver 43. These results indicate the reliability of the purification and proteomic techniques.

Furthermore, the full list of proteins was submitted to KEGG server (http://www.kegg.jp; *Mus musculus*) for classification analysis, with human disease‐related clusters not considered. Overall, the proteins belonging to the metabolism consisted of approximately one‐quarter of the total identified proteins (Fig. 3Bc), confirming that MT is an important organelle in charge of cell metabolism. Among those metabolic proteins, proteins involved in carbohydrate, lipid and amino acid metabolism constituted 16%, 23% and 16%, respectively, which shows that 55% of proteins related to metabolism were involved in the utilization of fuel molecules. Not surprisingly, the proteins involved in energy metabolism comprised 13% proteins in the metabolism, confirming the hosting role of mitochondria in energy producing.

### Significantly changed proteins revealed by comparative proteomic analysis of LDs and mitochondria from livers of mice at different temperatures

To identify the proteins altered significantly when housing temperature changes, a fold change cut‐off of > 1.2 for up‐regulation or < 0.833 for down‐regulation with a *P* value cut‐off of 0.1 was set. The top 10 up‐regulated and top 10 down‐regulated proteins were displayed if the number of differential proteins was more than 10. The results are summarized in Table 1 for mitochondrial proteins. In total, 77, 247 and 53 proteins showed obvious changes in the *T*_6_ vs. *T*_23_ group, *T*_6_ vs. *T*_30_ group and *T*_23_ vs. *T*_30_ group, respectively. When compared with the thermoneutral environment, the standard temperature can result in a significant change in mitochondrial protein expression. The number of the changed proteins in the *T*_6_ vs. *T*_30_ group was the greatest, which shows that mice need more changes to meet the energy demand during extreme cold temperatures. Table 2 summarizes the overall protein changes in LD. In total, 21, 39 and 9 proteins were screened as significant changed proteins in the *T*_6_ vs. *T*_23_ group, *T*_6_ vs. *T*_30_ group, and *T*_23_ vs. *T*_30_ group, respectively. Similar to mitochondrial proteins, more LD proteins in mouse liver show changes in a severe cold environment compared to in a moderate cold environment. The alteration of the two key metabolic organelles between 30 and 23 °C demonstrates that mice housed at 23 °C are under a cold stress condition. All of the significantly changed proteins are listed in Tables [Link], [Link].

**Table 1:** Significantly changed proteins revealed by comparative proteomic analysis of liver mitochondria from mice at different temperatures.

Groups	Pattern	Number of proteins	UniProtKB Accession Number	Protein description	Gene name	Ratio	*P* value
*T*_6_ vs. *T*_23_	Up‐regulated	18	Q14DH7	Acyl‐CoA synthetase short‐chain family member 3	Acss3	1.46	0.06
			Q91X75	Cyp2a4 protein	Cyp2a5	1.38	0.10
			O70400	PDZ and LIM domain protein 1	Pdlim1	1.36	0.07
			Q8K0L3	Acyl‐coenzyme A synthetase ACSM2	Acsm2	1.35	0.04
			Q505D7	Optic atrophy 3 protein homolog	Opa3	1.33	0.02
			P29758	Ornithine aminotransferase	Oat	1.32	0.01
			Q9WVM8	Kynurenine/alpha‐aminoadipate aminotransferase	Aadat	1.29	0.03
			Q9QZA0	Carbonic anhydrase 5B	Ca5b	1.25	0.07
			Q504M2	MCG53395	Pdp2	1.25	0.09
			D3YYS6	Monoglyceride lipase	Mgll	1.25	0.07
	Down‐regulated	59	Q8VCF0	Mitochondrial antiviral‐signalling protein	Mavs	0.34	0.09
			Q8BSE0	Regulator of microtubule dynamics protein 2	Rmdn2	0.39	0.06
			Q9CZW5	Mitochondrial import receptor subunit TOM70	Tomm70	0.52	0.06
			Q8BH80	Vesicle‐associated membrane protein, associated protein B and C	Vapb	0.52	0.10
			Q9WV55	Vesicle‐associated membrane protein‐associated protein A	Vapa	0.52	0.07
			Q3UJU9	Regulator of microtubule dynamics protein 3	Rmdn3	0.52	0.06
			F6U775	Dynamin‐like 120 kDa protein	Opa1	0.56	0.06
			P13516	Acyl‐CoA desaturase 1	Scd1	0.57	0.03
			O70303	Cell death activator CIDE‐B	Cideb	0.62	0.03
			Q8VDJ3	Vigilin	Hdlbp	0.63	0.02
*T*_6_ vs. *T*_30_	Up‐regulated	175	P70670	Nascent polypeptide‐associated complex subunit alpha	Naca	2.44	0.04
			Q9CXV1	Succinate dehydrogenase [ubiquinone] cytochrome *b* small subunit	Sdhd	2.23	0.02
			D3YVW2	Golgi integral membrane protein 4	Golim4	2.16	0.01
			P22599	Alpha‐1‐antitrypsin 1–2	Serpina1b	2.13	0.05
			Q8BWY3	Eukaryotic peptide chain release factor subunit 1	Etf1	1.97	0.10
			Q91X75	Cyp2a4 protein	Cyp2a5	1.95	0.003
			Q9ET30	Transmembrane 9 superfamily member 3	Tm9sf3	1.94	0.06
			Q3TGU7	Proliferation‐associated 2G4	Pa2 g4	1.93	0.01
			Q9JMG1	Endothelial differentiation‐related factor 1	Edf1	1.91	0.04
			Q9CZR8	Elongation factor Ts	Tsfm	1.79	0.01
	Down‐regulated	72	Q05816	Fatty acid‐binding protein, epidermal	Fabp5	0.36	0.004
			P63030	MPC 1	Mpc1	0.44	0.006
			Q9D0B5	Thiosulfate sulfurtransferase/rhodanese‐like domain‐containing protein 3	Tstd3	0.46	0.03
			Q8VCF0	Mitochondrial antiviral‐signalling protein	Mavs	0.51	0.07
			P62806	Histone H4	Hist1h4a	0.52	0.06
			A0A1W2P768	Histone H3.2	Hist2h3c1	0.55	0.10
			P62996	Transformer‐2 protein homolog beta	Tra2b	0.55	0.07
			A0A0N4SVP8	Predicted pseudogene 5580	Gm5580	0.56	0.01
			Q9D3D9	ATP synthase subunit delta	Atp5d	0.58	0.01
			Q62425	Cytochrome *c* oxidase subunit NDUFA4	Ndufa4	0.58	0.01
*T*_23_ vs. *T*_30_	Up‐regulated	34	Q9CXV1	Succinate dehydrogenase [ubiquinone] cytochrome *b* small subunit	Sdhd	1.83	0.08
			Q9ESP1	Stromal cell‐derived factor 2‐like protein 1	Sdf2 l1	1.61	0.09
			Q9CR21	Acyl carrier protein	Ndufab1	1.61	0.09
			P00186	Cytochrome P450 1A2	Cyp1a2	1.42	0.08
			Q99L04	Dehydrogenase/reductase SDR family member 1	Dhrs1	1.41	0.01
			Q91X77	Cytochrome P450 2C50	Cyp2c50	1.40	0.09
			Q5HZI9	Solute carrier family 25 member 51	Slc25a51	1.39	0.06
			G3X9F4	Transmembrane protein 143	Tmem143	1.39	0.06
			P61027	Ras‐related protein Rab‐10	Rab10	1.38	0.06
			Q9DC16	ER‐Golgi intermediate compartment protein 1	Ergic1	1.38	0.08
	Down‐regulated	19	P62806	Histone H4	Hist1h4a	0.40	0.05
			P10922	Histone H1.0	H1f0	0.41	0.07
			A0A1W2P768	Histone H3.2	Hist2h3c1	0.42	0.09
			P63030	MPC 1	Mpc1	0.56	0.00
			Q64433	10 kDa heat shock protein	Hspe1	0.63	0.06
			P09671	Superoxide dismutase [Mn]	Sod2	0.65	0.07
			A0A0N4SVP8	Predicted pseudogene 5580	Gm5580	0.68	0.04
			F8VPU2	FERM, ARHGEF and pleckstrin domain‐containing protein 1	Farp1	0.69	0.07
			P61458	Pterin‐4‐alpha‐carbinolamine dehydratase	Pcbd1	0.70	0.05
			P10852	4F2 cell‐surface antigen heavy chain	Slc3a2	0.70	0.03

**Table 2:** Significantly changed proteins revealed by comparative proteomic analysis of liver LDs from mice at different temperatures.

Groups	Pattern	Number of proteins	UniProtKB Accession Number	Protein description	Gene name	Ratio	*P* value
*T*_6_ vs. *T*_23_	Up‐regulated	21	B5X0G2	MUP 17	Mup17	1.72	0.06
			P35980	60S ribosomal protein L18	Rpl18	1.53	0.01
			Q6ZWN5	40S ribosomal protein S9	Rps9	1.53	0.08
			Q8BVZ1	Perilipin‐5	Plin5	1.46	0.06
			Q9DCM2	Glutathione *S*‐transferase kappa 1	Gstk1	1.43	0.01
			Q99LB2	Dehydrogenase/reductase SDR family member 4	Dhrs4	1.38	0.06
			Q99P30	Peroxisomal coenzyme A diphosphatase NUDT7	Nudt7	1.38	0.07
			P14131	40S ribosomal protein S16	Rps16	1.36	0.04
			Q01853	Transitional ER ATPase	Vcp	1.36	0.03
			E9QKR0	Guanine nucleotide‐binding protein G(I)/G(S)/G(T) subunit beta‐2	Gnb2	1.34	0.04
*T*_6_ vs. *T*_30_	Up‐regulated	29	B5X0G2	MUP 17	Mup17	2.32	0.02
			P25688	Uricase	Uox	1.81	0.01
			P32020	Non‐specific lipid‐transfer protein	Scp2	1.76	0.01
			P24270	Catalase	Cat	1.70	0.01
			Q4FZE8	MUP 1	Mup1	1.65	0.04
			Q9DCM2	Glutathione *S*‐transferase kappa 1	Gstk1	1.57	0.03
			Q99MZ7	Peroxisomal trans‐2‐enoyl‐CoA reductase	Pecr	1.56	0.02
			Q9WU19	Hydroxyacid oxidase 1	Hao1	1.53	0.04
			Q9JKR6	Hypoxia up‐regulated protein 1	Hyou1	1.46	0.04
			Q91WG0	Acylcarnitine hydrolase	Ces2c	1.44	0.04
	Down‐regulated	10	P12710	Fatty acid‐binding protein	Fabp1	0.74	0.09
			F7A8H6	Glutathione peroxidase	Gpx4	0.74	0.01
			P46638	Ras‐related protein Rab‐11B	Rab11b	0.77	0.02
			P43883	Perilipin‐2	Plin2	0.78	0.06
			Q8VCR2	17‐beta‐hydroxysteroid dehydrogenase 13	Hsd17b13	0.79	0.01
			P35279	Ras‐related protein Rab‐6A	Rab6a	0.80	0.01
			Q99JI6	Ras‐related protein Rap‐1b	Rap1b	0.80	0.03
			P62821	Ras‐related protein Rab‐1A	Rab1A	0.81	0.10
			Q9D1G1	Ras‐related protein Rab‐1B	Rab1b	0.82	0.05
			Q3TLP8	RAS‐related C3 botulinum substrate 1	Rac1	0.82	0.01
*T*_23_ vs. *T*_30_	Up‐regulated	2	A0A0R4J110	Iodothyronine deiodinase	Dio1	1.39	0.03
			P24456	Cytochrome P450 2D10	Cyp2d10	1.22	0.07
	Down‐regulated	7	A2AE89	Glutathione *S*‐transferase	Gstm1	0.58	0.07
			A0A1D5RMD4	Kinesin‐like protein KIF16B	Kif16b	0.72	0.08
			P46638	Ras‐related protein Rab‐11B	Rab11b	0.78	0.03
			Q3TLP8	RAS‐related C3 botulinum substrate 1	Rac1	0.78	0.003
			P56480	ATP synthase subunit beta	Atp5b	0.80	0.07
			P43883	Perilipin‐2	Plin2	0.80	0.08
			P84096	Rho‐related GTP‐binding protein RhoG	Rhog	0.81	0.05

### Pathway enrichment analysis using significantly changed proteins in mitochondria

Those differential proteins in mitochondria were further used to determine the enriched pathways using the KEGG database to provide insight into the cellular process affected by housing temperatures. Figure 3C shows that differential proteins enriched in the metabolic pathways were greatest in all three groups, indicating that metabolism was most affected by environmental temperatures. The amount of the differential proteins (69 proteins) involved in metabolic pathways was the greatest when comparing mice living at *T*_N_ with those in extreme cold conditions, indicating that mice had to increase the metabolic rate much more during conditions of extreme cold. In addition, the differential proteins related to carbohydrate, lipid and amino acid metabolism, the three main fuel molecules, differed greatly among the three groups, suggesting that possible changes in substrate selection as a fuel may take place at distinct temperatures. Ribosome proteins were also significantly enriched when comparing extreme cold conditions with thermoneutral conditions, which may provide a clue with respect to differences in liver mass between the two groups.

In addition to the above pathway enrichment analysis, the proteins enriched into pathways with *P* < 0.05 were further analyzed to obain a global view of how they interact and form cellular networks to influence cellular processes. The STRING database was adopted to map the interaction (https://string-db.org). Several apparent interaction groups exerting diverse biological functions were displayed when the housing temperature changed (Fig. S1). The most complicated networks were formed in the *T*_6_ vs. *T*_30_ group, suggesting that most changes would take place when the environment was extremely cold, which is consistent with the above results. Proteins involved in oxidative phosphorylation, ribosomes, the TCA cycle, retinol metabolism and steroid hormone biosynthesis form the main association networks, which revealed a significant influence on these intracellular processes as a result of exposure to cold. Proteins involved in steroid hormone biosynthesis form an interaction in all three groups, suggesting steroid hormone metabolism was particularly sensitive to cold.

### Pathways over‐represented or under‐represented

**Figure 4:**
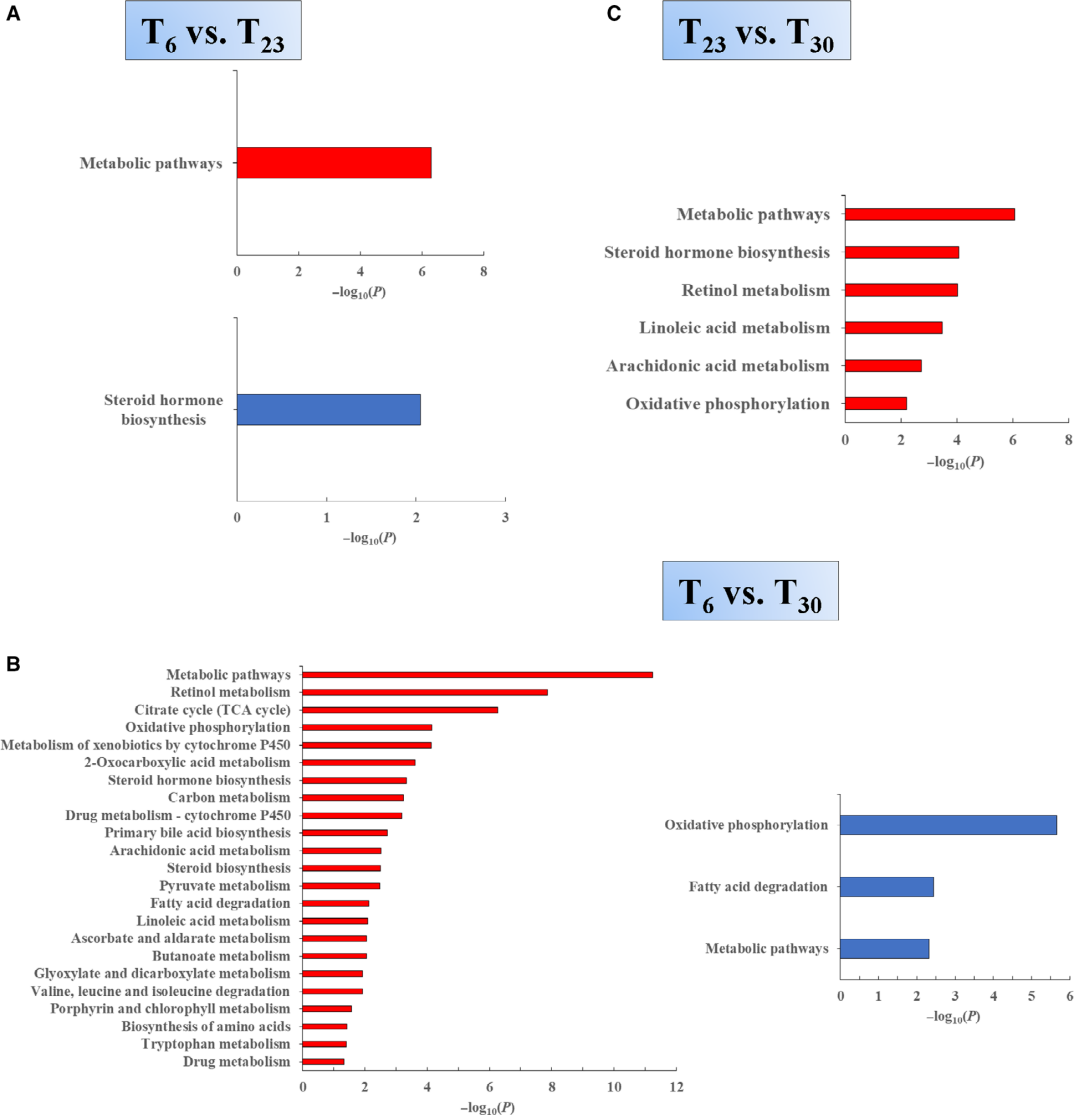
Metabolic pathways over‐represented or under‐represented in the liver mitochondria from mice living at different temperatures. The identified proteins that were up‐ or down‐regulated in the liver mitochondria under different conditions were analyzed using the KEGG database for over‐ or under‐represented pathways involved in metabolism. The bars represent −log_10_(*P*; the enrichment factors). Significantly affected pathways should have −log_10_ (*P*) value score > 1.3 (*P* < 0.05). Red bars or blue bars represent the pathway −log_10_ (*P*) value score calculated using only up‐regulated proteins or down‐regulated proteins, respectively. Metabolic pathways with at least three differential proteins identified are shown. Under these criteria, over‐ or under‐represented metabolic pathways from the *T*_6_ vs. *T*_23_ (A), *T*_6_ vs. *T*_30_ (B) and *T*_23_ vs. *T*_30_ (C) comparations are shown.

Because metabolism was obviously affected by environmental temperature, we further analyzed the metabolic pathways that were over‐ or under‐represented, aiming to obtain information on how the mouse liver adapts in various environmental temperatures. To enhance the reliability of the bioinformatic analysis, significantly affected pathways with a *P* value cut‐off of 0.05 and with at least three differential proteins were considered. Under these criteria, in the *T*_6_ vs. *T*_23_ group, the steroid hormone biosynthesis process was under‐represented in the *T*_6_ group (Fig. 4A). In the *T*_6_ vs. *T*_30_ group, several pathways were over‐represented in the *T*_6_ group (Fig. 4B).

**Figure 5:**
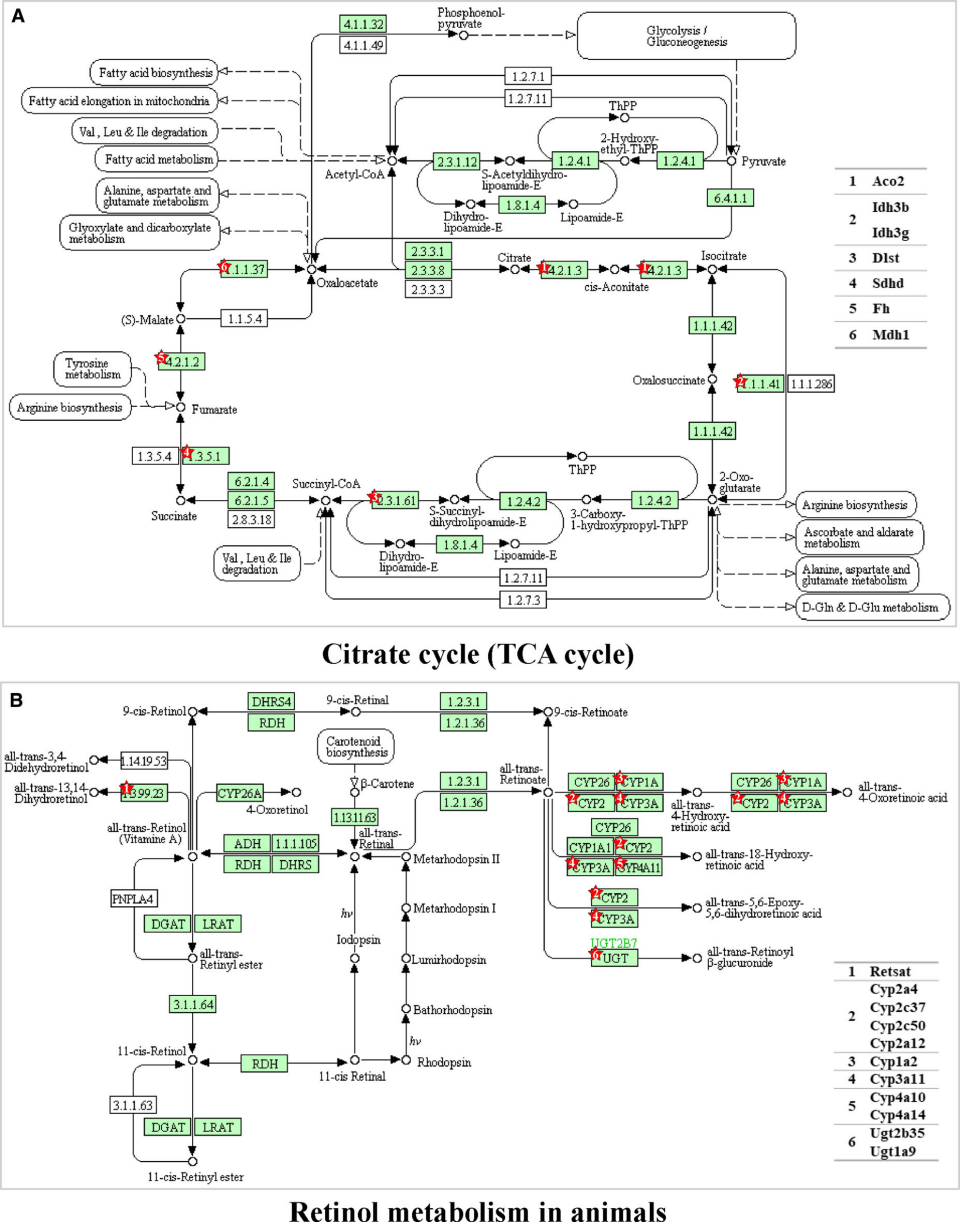
KEGG pathways for TCA cycle and retinol metabolism in the *T*_6_ vs. *T*_30_ group. In the *T*_6_ vs. *T*_30_ group, TCA cycle and retinol metabolism in mitochondria were enriched as over‐represented pathways. The list of proteins that were up‐regulated from proteomic analysis was further analyzed in the TCA cycle pathway (A) and retinol metabolism pathway (B) using the DAVID bioinformatics database (https://david.ncifcrf.gov/home.jsp). The red stars indicate the site where the differential proteins function.

As described above, our analysis revealed compelling changes of proteins involved in TCA cycle and retinol metabolism in the *T*_6_ vs. *T*_30_ group (Figs 3 and S1). Further analysis showed that retinol metabolism pathway and the TCA cycle are over‐represented in the liver mitochondria of mice living at a temperature of 6 °C (Fig. 4B). Retinol and its metabolites are shown to be involved in the regulation of metabolism in the liver and whole body. Eleven significantly changed proteins were involved in retinol metabolism and they were all up‐regulated when comparing *T*_6_ with *T*_30_. These proteins include all‐trans‐retinol 13,14‐reductase, two UDP‐glucuronosyltransferases and eight proteins from the cytochrome P450 family. The pathway and those proteins were analyzed by the DAVID database (https://david.ncifcrf.gov/home.jsp) and are shown in Fig. 5. Seven significantly changed proteins were involved in the TCA cycle and they were all up‐regulated when comparing *T*_6_ with *T*_30_. These proteins include aconitate hydratase, two isocitrate dehydrogenase, dihydrolipoyllysine‐residue succinyltransferase component of 2‐oxoglutarate dehydrogenase complex, succinate dehydrogenase, fumarate hydratase and malate dehydrogenase. The pathway and those proteins were analyzed by DAVID the bioinformatics database (https://david.ncifcrf.gov/home.jsp) and are shown in Fig. 5.

In both the *T*_6_ vs. *T*_23_ group and the *T*_6_ vs. *T*_30_ group, oxidative phosphorylation was not enriched as an over‐ or under‐represented pathway (Fig. 4A,B). This result suggested that oxidative phosphorylation was not affected after 4 weeks of cold exposure, as reported previously 44. The TCA cycle functions to degrade acetyl‐CoA into CO_2_, yielding reducing power to be used in oxidative phosphorylation to produce ATP, and also to provide intermediates for biosynthetic processes. Under extremely cold conditions, the TCA cycle was shown to be enhanced, whereas oxidative phosphorylation showed no obvious change, hence suggesting that liver mitochondria may act to provide biosynthetic substances to help liver to adapt.

In the *T*_23_ vs. *T*_30_ group, no under‐represented pathways in the *T*_23_ group were enriched, whereas steroid hormone biosynthesis and retinol metabolism pathways were over‐represented in the *T*_23_ group (Fig. 4C). All of the enriched pathways are listed in Table S3.

### Verification of the proteomic results

**Figure 6:**
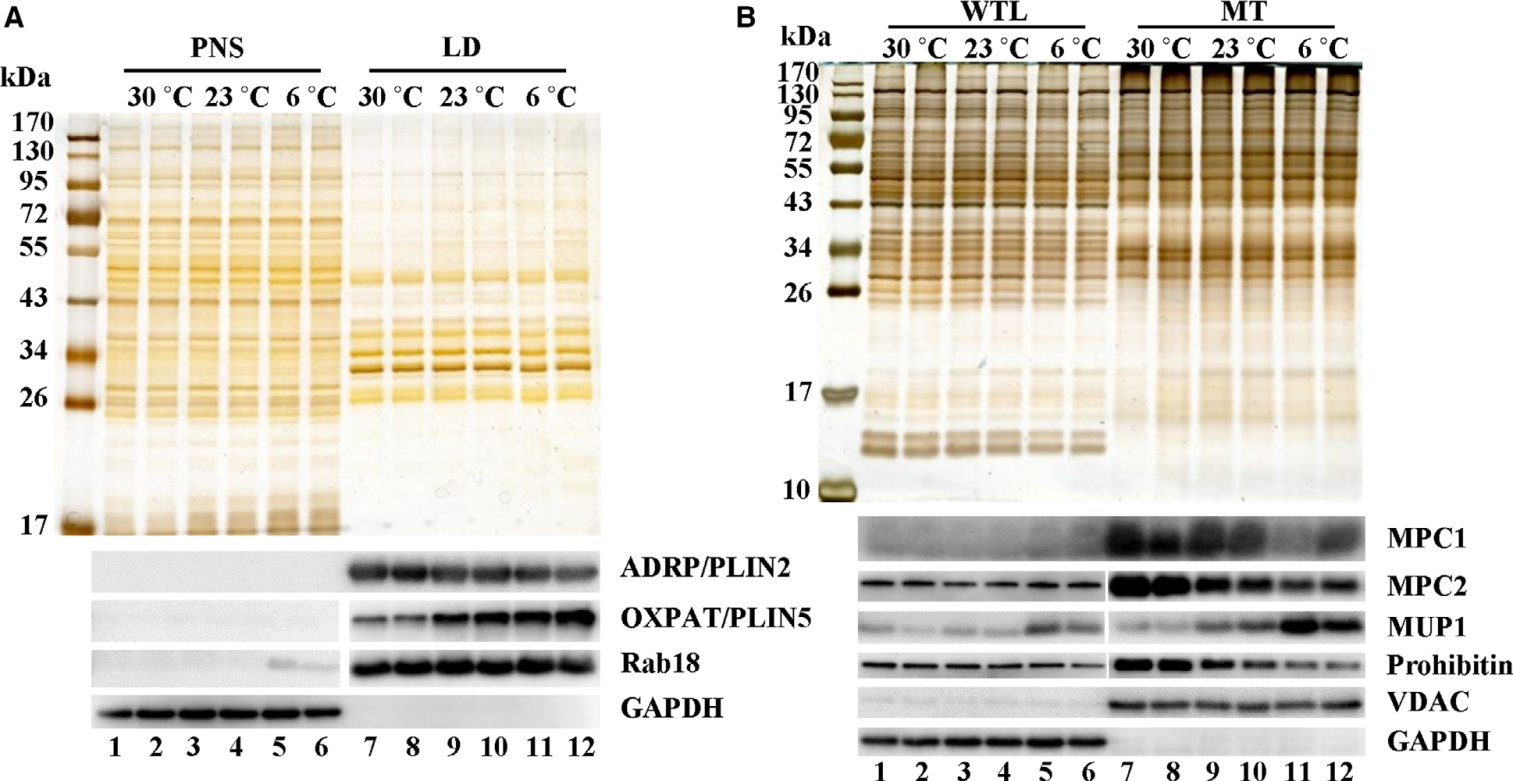
Verification of proteomic results by western blotting. The expression of several proteins on isolated LDs and mitochondria was tested by western blotting to verify the accuracy of the TMT‐based quantification. (A) Verification of the expression pattern of LD proteins from three conditions. The LD proteins PLIN2/ADRP, PLIN5/OXPAT and Rab18 were tested by western blotting (lower) with equal protein loading shown by silver staining (upper). (B) Verification of the expression pattern of mitochondrial proteins from three conditions. WTL, whole tissue lysate. The proteins MPC1, MPC2, MUP1, Prohibitin and VDAC were tested by western blotting (lower) with equal protein loading shown by silver staining (upper).

To confirm the TMT‐based comparative quantification results, immunoblotting analysis was conducted. In agreement with the proteomic study, the expression of LD resident protein, PLIN2/ADRP was decreased, whereas PLIN5 increased when the housing temperature decreased (Fig. 6A). The quantification results were also verified by the expression pattern of protein Rab18 determined using immunoblotting.

Several proteins were also selected for the verification of the mitochondrial quantification results (Fig. 6B). Expression of MPC, MPC1 and MPC2 was decreased when the environmental temperature decreased. MUP1 in mitochondria was identified as a protein that increased dramatically when the mouse was exposed to cold. Immunoblotting analysis indeed showed that the expression of MUP1 was enhanced significantly under extreme cold conditions. VDAC showed no obvious change, whereas prohibitin was decreased. The data show that a reliable proteomic database for liver LDs and mitochondria from mouse living at different temperatures has been set up. The mass spectrometry proteomics data have been deposited in the ProteomeXchange Consortium via the PRIDE 45 partner repository with the dataset identifier PXD014224.

## Discussion

Mammals try to maintain a constant core temperature in diverse environments. Humans choose an easier way of meeting the goal by wearing clothing and/or using air‐conditioners to regulate the ambient temperature. However, this condition set the laboratory mouse under chronic cold stress. When laboratory mice were housed at different temperatures, they regulated their body to adapt to distinct temperatures. For example, under extreme cold conditions, an increase in body weight was shown (Fig. 1). Liver mass showed an increase when the environmental temperature was lower than their *T*_N_ (i.e. standard temperature or extreme cold temperature). These results confirm that the change in environmental temperature will have a profound influence on the metabolism in the whole body and individual tissues. Because we always aim to live within our thermal comfort via our clothing and/or by changing the surrounding temperature, when housing our model animals, the environmental temperature should be considered seriously. Indeed, thermoneutral housing is required to model diet‐induced obesity in C57BL/6 nude mice 12. Housing at thermoneutrality would initiate atherosclerosis in wild‐type C57BL/6 mice 13. Giles *et al*. 10 found that thermoneutral housing exacerbated mouse nonalcoholic fatty liver disease and allowed for the development of a more ‘human‐like’ model. Therefore, housing laboratory mice at thermoneutrality is proposed to be an easier and better strategy for modelling human diseases and energy metabolism 9, 46.

The liver is one of the largest and most metabolically active organs in mammals. However, how the liver adapts itself at different temperatures remains unclear. Subsequently, we conducted a liver organelle proteomic study to investigate the involvement of LD and MT, two major metabolic organelles, when mice are raised at different temperatures. According to the LD comparative proteomic results, PLIN5 was screened in terms of obviously changed proteins showing the highest expression under extreme cold conditions. This was verified by immunoblotting (Fig. 6A). PLIN5 plays a central role in lipid homeostasis and is clearly required to limit the production of lipid intermediates and prevent disruption to tissue function 47. PLIN5 may play a role in the regulation of lipid metabolism in the mouse liver when mice are exposed to chronic cold stress.

Eukaryotic cells must organize cellular metabolism well to adapt the status of environmental situations and hence they exhibit outstanding plasticity in bioenergetics. In the present study, we found that liver TAG would increase under *T*_30_ housing compared to *T*_23_ housing, although with no change in histology (Fig. 1). Liver glycogen content showed no obvious change under these two conditions. Small *et al*. 48 found the abundance of key proteins involved in liver lipogenesis was reported to be elevated, whereas the rate‐limiting enzyme of gluconeogenesis showed no alteration in mice housed at *T*_N_ for 13 weeks. Under *T*_N_, the energy requirement for maintaining body temperature is reduced. Therefore, the liver would accumulate energy as occurred for the lipid under *T*_N_.

When chronically exposed to cold, profound systemic metabolic changes take place to enable the organism to adapt to the environmental thermal challenge 49. Cold exposure triggers energy expenditure. The current cellular metabolic patterns in mammals have evolved through the selective pressures of starvation and cold exposure 15. In mammals, BAT is an organ known to play a major role in protecting against cold through non‐shivering thermogenesis 50. During cold exposure, glucose uptake in human BAT is increased by 12‐fold 51. Besides carbohydrates, activation of BAT by cold exposure increased the utilization of fatty acids from triglyceride‐rich lipoproteins and fatty acids released by white adipocytes 52, 53. These studies suggest that BAT would input energy fuels, glucose and fatty acids from other organs, such as the liver, skeletal muscle and white adipose tissue, under cold exposure to help maintain thermogenesis 54.

Short‐term cold exposure and long‐term cold exposure elicit different liver responses 44. Because laboratory mice are always kept under chronic cold conditions, we investigated how the liver reacts and helps the body to adapt to long‐term cold exposure. Our results showed that, when confronted with chronic extreme cold, the C57BL/6 mouse would decrease liver glycogen significantly. In addition to the decrease of liver glycogen, 4‐week cold‐acclimated rats were reported to show a decrease of white adipose tissue mass by 20% compared to rats housed at a standard temperature 55. Furthrmore, their plasma glucose and cholesterol esters were not affected either by exposure to an extreme cold temperature. However, plasma TAG was obviously deceased. More importantly, it has been shown that the liver in cold acclimated rats has an increased capacity for gluconeogenesis 56, 57. The incorporation of tritium from ^3^H_2_O into liver fatty acids was elevated 2.2‐fold in 4‐week cold‐acclimated rats compared to warm‐acclimated rats 58. Liver from rats living under cold conditions (1–2 °C) for 14 days showed a decrease with respect to converting radiolabelled glucose into CO_2_ compared to that from rats housed at 25 °C 59. Hepatic mitochondrial oxidative phosphorylation showed no change after rats were exposed to cold (4 °C) for 30 days 44.

According to our comparative proteomic analysis of MT, when the surrounding temperature was lower, the mouse acted to enhance the mitochondrial TCA cycle in the liver (Fig. 4B). However, in agreement with previous results 44, hepatic oxidative phosphorylation under long‐term cold acclimation was not obviously affected (Fig. 4A,B). Elevated TCA cycle flux was reported to be linked to the increase in gluconeogenesis and lipogenesis from TCA cycle precursors 20, 60. Hence, the liver mitochondria may increase the TCA cycle capacity to increase biosynthetic processes such as gluconeogenesis and lipogenesis, aiming to help the liver and whole body to adapt to long‐term cold exposure.

Another point for consideration is how carbohydrate and fatty acid metabolism change under chronic extreme cold situations. Pyruvate is a key metabolite of glucose, the major simple carbohydrate. Our proteomic results showed a significant decrease of the abundance of MPC in the liver living at *T*_6_, which was further verified by immunoblotting analysis (Fig. 6B). MPC, a mitochondrial inner member protein, transports pyruvate from the cytosol to the mitochondrial matrix and thus acts as a central node connecting carbohydrate, amino acid and fatty acid metabolism 21, 61. Is the decrease of MPC related to the change in metabolism in the liver of cold‐acclimated mice?

A deficiency in MPC activity displayed a defect in glucose‐derived pyruvate oxidation 61, 62. However, disruption of MPC activity was reported to promote fatty acid oxidation and glutamine oxidation to sustain TCA cycle flux, as well as to increase lipogenesis 61, 63, 64, 65, 66. In addition, a loss of MPC in the mouse liver resulted in reduced pyruvate‐driven gluconeogenesis, which could be compensated for via pyruvate‐alanine cycling 64, 65. Therefore, inhibition of mitochondrial pyruvate uptake (i.e. a reduction in glucose‐derived pyruvate oxidation) was proposed to be able to rewrite the cellular metabolism 61.

Based on the results obtained in the present study, as well as those of previous studies mentioned above, we propose a hypothesis concerning the role of the liver in adaptive metabolic responses when the mouse is confronted with extreme cold condition. When the environmental temperature is extremely cold, the liver degrades glycogen and increases gluconeogenesis to release glucose to maintain whole body glucose. Furthrmore, the liver may shut down carbohydrate oxidation by turning off pyruvate transport into the MT. The reduction in pyruvate‐derived gluconeogenesis would be compensated for by tissue cross‐talk. For example, glycerol released from adipose tissue and lactic acid from muscle may be taken by liver to produce glucose. In addition, the liver may increase the mitochondrial TCA cycle capacity to increase biosynthetic processes, such as gluconeogenesis and lipogenesis. Accordingly, the liver changes its own metabolism to adapt to chronic cold stress and maintain blood glucose levels at the same time.

Noticeably, MUP17 and MUP1 were the most highly elevated proteins in the LD proteome when the environmental temperature was decreased in both the *T*_6_ vs. *T*_23_ group and the *T*_6_ vs. *T*_30_ group. According to the results of western blotting, MUP1 in mitochondria also showed a dramatic increase when the housing temperature decreased (Fig. 6B). MUPs comprise lipocalin family members synthesized predominantly in the liver and secreted into the circulation. The MUPs in the mouse are excreted into urine and account for more than 90% proteins in the urine 67, 68. MUPs in the mouse are traditionally proposed to act as a pheromone‐binding protein in urine, playing a key role in chemical communication 69, 70. In addition, MUPs themselves can function as a protein pheromone to facilitate chemical information exchange 70. In 2009, it was shown that MUP1 comprises a humoral metabolic regulator in mice 71 and that chronic elevation of circulating MUP1 in *db/db* mice could increase energy expenditure and raise the core body temperature. Subsequently, additional studies showed that MUPs are involved in the regulation of hepatic gluconeogenic and lipid metabolism 72. In addition, MUPs belong to the lipocalin superfamily, sharing the characteristic eight β‐strands forming a barrel to bind and transport small hydrophobic molecules, including steroid hormones, retinoids, odorants (e.g. pheromones) and lipids 71, 72. Lipocalin proteins play important roles in physiological processes by transporting molecules. For example, lipocalin 2 was recently reported to be a new adipose‐derived cytokine and to play a critical role in regulating retinoid homeostasis and retinoid‐mediated thermogenesis in adipose tissue 73, 74.

The liver is the main site for the storage of retinyl ester. We found that the retinyl ester in the liver of mice living under a cold environment increased. Our proteomic data showed that, under a cold environment, the retinol metabolism in liver mitochondria was over‐represented significantly. In addition, retinoids are known to be important physiological regulators of thermogenesis 75. The requirements for retinoids were increased in the cold and vitamin A deficiency would lead to a reduced survival time for rats 76. Therefore, MUP may be a metabolic regulator in the mouse liver by regulating retinol metabolism when mice were exposed to chronic cold.

As mentioned above, MUPs are mainly synthesized in the liver and secreted into serum. Because it is hard to exclude the existence of trace amounts of ER in isolated mitochondria, we analyzed the MUP1 distribution in the cellular component to determine whether the detection of MUP1 in mitochondria was a result of the mitochondrial associated ER. After cell fractionation, MUP1 distribution was analyzed by western blotting. With a similar amount of ER marker protein in mitochondrial and TM fractions, more MUP1 was detected in isolated mitochondria (Fig. S2). This result indicates that the detection of MUP1 in isolated mitochondria was not a result of contact between mitochondria and ER. Indeed, similar to our results, MUP1 was also found in isolated liver mitochondria via immunoblotting in another study 77. Besides, MUP1 has been reported to enhance mitochondrial biogenesis 71. Interestingly, when the environmental temperature decreased, we also observed a dramatic increase in the expression of PGC‐1α, a major regulator of mitochondrial biogenesis 78, in the mouse liver (Fig. S2). Thus, MUP1 may be dynamically localized to mitochondria to help the liver to adapt when mice are confronted with cold conditions.

In addition, because MUP1 is secreted into serum, we also tested serum MUP1 in mice living at different temperatures. Circulating MUP1 was obviously enhanced in mice living under a cold temperature (Fig. S2). Impressively, lipocalin 2 was reported to be able to regulate BAT activation via a nonadrenergic pathway 73. Both MUP1 and lipocalin 2 belong to the lipocalin superfamily. We compared their structures and the results obtained demonstrated that they showed a high degree of structural similarity (Fig. S2). MUP1 may play a role similar to that of lipocalin 2 when the environment is cold. Certainly, the biological function of MUPs requires further investigation.

## Summary

The liver acts as a hub with respect to controlling whole‐body metabolism. The present study has revealed the characteristics of liver LD and MT of laboratory mice living in their *T*_N_ because they are always set in a cold room, resulting in metabolic alterations in the liver. Furthermore, the present study highlights the metabolic changes in the liver, an organ that is not taken seriously under conditions of exposure to chronic cold. We found that MPC, MUPs and PLIN5 may play a role in liver adaptation in laboratory mice living under chronic cold conditions.

## Author contributions

SZ and PL designed the project. QL and SZ performed the experiments. ZZ helped with the bioinformatic analysis. SZ and PL wrote the manuscript. All authors have read and approved the final version of the manuscript submitted for publication.

## Supporting information


**Fig. S1**. The association network of differential proteins in the liver mitochondria from mice living at different temperatures.

**Fig. S2**. Subcellular localization of MUP1 and its possible function in mice living in a cold environment.

**Table S1**. Categorization of the identified proteins and significantly changed proteins of liver LDs from mice at different temperatures.

**Table S2**. List of the identified proteins and significantly changed proteins of liver mitochondria from mice at different temperatures.

**Table S3**. Pathways over‐represented or under‐represented in liver mitochondria from mice at different temperatures.

**Appendix S1**. Supplemental information for mass spectrometry and Figs S1 and S2.

## References

[1] Karp CL (2012) Unstressing intemperate models: how cold stress undermines mouse modeling. J Exp Med 209, 1069–1074. 22665703 10.1084/jem.20120988 PMC3371737

[2] Maloney SK , Fuller A , Mitchell D , Gordon C and Overton JM (2014) Translating animal model research: does it matter that our rodents are cold? Physiology (Bethesda) 29, 413–420. 25362635 10.1152/physiol.00029.2014

[3] Lodhi IJ and Semenkovich CF (2009) Why we should put clothes on mice. Cell Metab 9, 111–112. 19187768 10.1016/j.cmet.2009.01.004

[4] Cannon B and Nedergaard J (2011) Nonshivering thermogenesis and its adequate measurement in metabolic studies. J Exp Biol 214 (Pt 2), 242–253. 21177944 10.1242/jeb.050989

[5] Nedergaard J and Cannon B (2014) The browning of white adipose tissue: some burning issues. Cell Metab 20, 396–407. 25127354 10.1016/j.cmet.2014.07.005

[6] Erikson H , Krog J , Andersen KL and Scholander PF (1956) The critical temperature in naked man. Acta Physiol Scand 37, 35–39. 13339450 10.1111/j.1748-1716.1956.tb01339.x

[7] Scholander PF , Andersen KL , Krog J , Lorentzen FV and Steen J (1957) Critical temperature in lapps. J Appl Physiol 10, 231–234. 13428651 10.1152/jappl.1957.10.2.231

[8] Hill RW , Muhich TE and Humphries MM (2013) City‐scale expansion of human thermoregulatory costs. PLoS ONE 8, e76238. 24143181 10.1371/journal.pone.0076238 PMC3797062

[9] Ganeshan K and Chawla A (2017) Warming the mouse to model human diseases. Nat Rev Endocrinol 13, 458–465. 28497813 10.1038/nrendo.2017.48 PMC5777302

[10] Giles DA , Moreno‐Fernandez ME , Stankiewicz TE , Graspeuntner S , Cappelletti M , Wu D , Mukherjee R , Chan CC , Lawson MJ , Klarquist J *et al* (2017) Thermoneutral housing exacerbates nonalcoholic fatty liver disease in mice and allows for sex‐independent disease modeling. Nat Med 23, 829–838. 28604704 10.1038/nm.4346 PMC5596511

[11] Rudaya AY , Steiner AA , Robbins JR , Dragic AS and Romanovsky AA (2005) Thermoregulatory responses to lipopolysaccharide in the mouse: dependence on the dose and ambient temperature. Am J Physiol Regul Integr Comp Physiol 289, R1244–R1252. 16081879 10.1152/ajpregu.00370.2005

[12] Stemmer K , Kotzbeck P , Zani F , Bauer M , Neff C , Müller TD , Pfluger PT , Seeley RJ and Divanovic S (2015) Thermoneutral housing is a critical factor for immune function and diet‐induced obesity in C57BL/6 nude mice. Int J Obes (Lond) 39, 791–797. 25349057 10.1038/ijo.2014.187 PMC4412759

[13] Giles DA , Ramkhelawon B , Donelan EM , Stankiewicz TE , Hutchison SB , Mukherjee R , Cappelletti M , Karns R , Karp CL , Moore KJ *et al* (2016) Modulation of ambient temperature promotes inflammation and initiates atherosclerosis in wild type C57BL/6 mice. Mol Metab 5, 1121–1130. 27818938 10.1016/j.molmet.2016.09.008 PMC5081423

[14] Tian XY , Ganeshan K , Hong C , Nguyen KD , Qiu Y , Kim J , Tangirala RK , Tontonoz P and Chawla A (2016) Thermoneutral housing accelerates metabolic inflammation to potentiate atherosclerosis but not insulin resistance. Cell Metab 23, 165–178. 26549485 10.1016/j.cmet.2015.10.003 PMC4715491

[15] Simcox J , Geoghegan G , Maschek JA , Bensard CL , Pasquali M , Miao R , Lee S , Jiang L , Huck I , Kershaw EE *et al* (2017) Global analysis of plasma lipids identifies liver‐derived acylcarnitines as a fuel source for brown fat thermogenesis. Cell Metab 26, 509–522.e6. 28877455 10.1016/j.cmet.2017.08.006 PMC5658052

[16] Farese RV Jr and Walther TC (2009) Lipid droplets finally get a little R‐E‐S‐P‐E‐C‐T. Cell 139, 855–860. 19945371 10.1016/j.cell.2009.11.005 PMC3097139

[17] Zehmer JK , Huang Y , Peng G , Pu J , Anderson RG and Liu P (2009) A role for lipid droplets in inter‐membrane lipid traffic. Proteomics 9, 914–921. 19160396 10.1002/pmic.200800584 PMC2676673

[18] Zhang S , Wang Y , Cui L , Deng Y , Xu S , Yu J , Cichello S , Serrero G , Ying Y and Liu P (2016) Morphologically and functionally distinct lipid droplet subpopulations. Sci Rep 6, 29539. 27386790 10.1038/srep29539 PMC4937419

[19] Samuel VT and Shulman GI (2012) Mechanisms for insulin resistance: common threads and missing links. Cell 148, 852–871. 22385956 10.1016/j.cell.2012.02.017 PMC3294420

[20] Owen OE , Kalhan SC and Hanson RW (2002) The key role of anaplerosis and cataplerosis for citric acid cycle function. J Biol Chem 277, 30409–30412. 12087111 10.1074/jbc.R200006200

[21] Bender T and Martinou JC (2016) The mitochondrial pyruvate carrier in health and disease: to carry or not to carry? Biochim Biophys Acta 1863, 2436–2442. 26826034 10.1016/j.bbamcr.2016.01.017

[22] Ding Y , Zhang S , Yang L , Na H , Zhang P , Zhang H , Wang Y , Chen Y , Yu J , Huo C *et al* (2013) Isolating lipid droplets from multiple species. Nat Protoc 8, 43–51. 23222457 10.1038/nprot.2012.142

[23] Yu J , Zhang S , Cui L , Wang W , Na H , Zhu X , Li L , Xu G , Yang F , Christian M *et al* (2015) Lipid droplet remodeling and interaction with mitochondria in mouse brown adipose tissue during cold treatment. Biochem Biophys Acta 1853, 918–928. 25655664 10.1016/j.bbamcr.2015.01.020

[24] Villarin JJ , Schaeffer PJ , Markle RA and Lindstedt SL (2003) Chronic cold exposure increases liver oxidative capacity in the marsupial *Monodelphis domestica* . Comp Biochem Physiol A Mol Integr Physiol 136, 621–630. 14613790 10.1016/s1095-6433(03)00210-1

[25] Zhao ZJ , Chi QS , Liu QS , Zheng WH , Liu JS and Wang DH (2014) The shift of thermoneutral zone in striped hamster acclimated to different temperatures. PLoS ONE 9, e84396. 24400087 10.1371/journal.pone.0084396 PMC3882234

[26] Hong Y , Li S , Wang J and Li Y (2018) *In vitro* inhibition of hepatic stellate cell activation by the autophagy‐related lipid droplet protein ATG2A. Sci Rep 8, 9232. 29915313 10.1038/s41598-018-27686-6 PMC6006255

[27] Vance JE (2014) MAM (mitochondria‐associated membranes) in mammalian cells: lipids and beyond. Biochim Biophys Acta 1841, 595–609. 24316057 10.1016/j.bbalip.2013.11.014

[28] Na H , Zhang P , Ding Y , Yang L , Wang Y , Zhang H , Xie Z , Yang F , Cichello S and Liu P (2013) Proteomic studies of isolated lipid droplets from bacteria, *C. elegans*, and mammals. Methods Cell Biol 116, 1–14. 24099284 10.1016/B978-0-12-408051-5.00001-2

[29] Wang W , Wei S , Li L , Su X , Du C , Li F , Geng B , Liu P and Xu G (2015) Proteomic analysis of murine testes lipid droplets. Sci Rep 5, 12070. 26159641 10.1038/srep12070 PMC4498221

[30] Su W , Wang Y , Jia X , Wu W , Li L , Tian X , Li S , Wang C , Xu H , Cao J *et al* (2014) Comparative proteomic study reveals 17beta‐HSD13 as a pathogenic protein in nonalcoholic fatty liver disease. Proc Natl Acad Sci USA 111, 11437–11442. 25028495 10.1073/pnas.1410741111 PMC4128098

[31] Yu J , Zhang L , Li Y , Zhu X , Xu S , Zhou XM , Wang H , Zhang H , Liang B and Liu P (2018) The adrenal lipid droplet is a new site for steroid hormone metabolism. Proteomics 18, e1800136. 30358111 10.1002/pmic.201800136

[32] Walther TC and Farese RV Jr (2012) Lipid droplets and cellular lipid metabolism. Annu Rev Biochem 81, 687–714. 22524315 10.1146/annurev-biochem-061009-102430 PMC3767414

[33] Fujimoto Y , Itabe H , Sakai J , Makita M , Noda J , Mori M , Higashi Y , Kojima S and Takano T (2004) Identification of major proteins in the lipid droplet‐enriched fraction isolated from the human hepatocyte cell line HuH7. Biochim Biophys Acta 1644, 47–59. 14741744 10.1016/j.bbamcr.2003.10.018

[34] Liu P , Bartz R , Zehmer JK , Ying Y and Anderson RG (2008) Rab‐regulated membrane traffic between adiposomes and multiple endomembrane systems. Methods Enzymol 439, 327–337. 18374175 10.1016/S0076-6879(07)00424-7 PMC2649762

[35] Xu D , Li Y , Wu L , Li Y , Zhao D , Yu J , Huang T , Ferguson C , Parton RG , Yang H *et al* (2018) Rab18 promotes lipid droplet (LD) growth by tethering the ER to LDs through SNARE and NRZ interactions. J Cell Biol 217, 975–995. 29367353 10.1083/jcb.201704184 PMC5839781

[36] Wan HC , Melo RC , Jin Z , Dvorak AM and Weller PF (2007) Roles and origins of leukocyte lipid bodies: proteomic and ultrastructural studies. FASEB J 21, 167–178. 17135363 10.1096/fj.06-6711com PMC2715426

[37] Yang L , Ding Y , Chen Y , Zhang S , Huo C , Wang Y , Yu J , Zhang P , Na H , Zhang H *et al* (2012) The proteomics of lipid droplets: structure, dynamics, and functions of the organelle conserved from bacteria to humans. J Lipid Res 53, 1245–1253. 22534641 10.1194/jlr.R024117 PMC3371236

[38] Ohsaki Y , Cheng J , Suzuki M , Fujita A and Fujimoto T (2008) Lipid droplets are arrested in the ER membrane by tight binding of lipidated apolipoprotein B‐100. J Cell Sci 121 (Pt 14), 2415–2422. 18577578 10.1242/jcs.025452

[39] Jo Y , Hartman IZ and DeBose‐Boyd RA (2013) Ancient ubiquitous protein‐1 mediates sterol‐induced ubiquitination of 3‐hydroxy‐3‐methylglutaryl CoA reductase in lipid droplet‐associated endoplasmic reticulum membranes. Mol Biol Cell 24, 169–183. 23223569 10.1091/mbc.E12-07-0564 PMC3564538

[40] Bersuker K and Olzmann JA (2017) Establishing the lipid droplet proteome: mechanisms of lipid droplet protein targeting and degradation. Biochim Biophys Acta Mol Cell Biol Lipids 1862(10 Pt B), 1166–1177. 28627435 10.1016/j.bbalip.2017.06.006 PMC5595636

[41] Welte MA (2015) Expanding roles for lipid droplets. Curr Biol 25, R470–R481. 26035793 10.1016/j.cub.2015.04.004 PMC4452895

[42] Calvo SE , Clauser KR and Mootha VK (2016) Mootha, MitoCarta2.0: an updated inventory of mammalian mitochondrial proteins. Nucleic Acids Res 44 (D1), D1251–D1257. 26450961 10.1093/nar/gkv1003 PMC4702768

[43] Guo Y , Darshi M , Ma Y , Perkins GA , Shen Z , Haushalter KJ , Saito R , Chen A , Lee YS , Patel HH *et al* (2013) Quantitative proteomic and functional analysis of liver mitochondria from high fat diet (HFD) diabetic mice. Mol Cell Proteomics 12, 3744–3758. 24030101 10.1074/mcp.M113.027441 PMC3861721

[44] Liverini G , Iossa S and Barletta A (1992) Rat liver response elicited by long‐term cold exposure. J Physiol Paris 86, 195–200. 1343605 10.1016/0928-4257(92)90006-2

[45] Perez‐Riverol Y , Csordas A , Bai J , Bernal‐Llinares M , Hewapathirana S , Kundu DJ , Inuganti A , Griss J , Mayer G , Eisenacher M *et al* (2019) The PRIDE database and related tools and resources in 2019: improving support for quantification data. Nucleic Acids Res 47 (D1), D442–D450. 30395289 10.1093/nar/gky1106 PMC6323896

[46] Fischer AW , Cannon B and Nedergaard J (2018) Optimal housing temperatures for mice to mimic the thermal environment of humans: an experimental study. Mol Metab 7, 161–170. 29122558 10.1016/j.molmet.2017.10.009 PMC5784327

[47] Mason RR and Watt MJ (2015) Unraveling the roles of PLIN5: linking cell biology to physiology. Trends Endocrinol Metab 26, 144–152. 25682370 10.1016/j.tem.2015.01.005

[48] Small L , Gong H , Yassmin C , Cooney GJ and Brandon AE (2018) Thermoneutral housing does not influence fat mass or glucose homeostasis in C57BL/6 mice. J Endocrinol 239, 313–324. 30400016 10.1530/JOE-18-0279

[49] Brychta RJ and Chen KY (2017) Cold‐induced thermogenesis in humans. Eur J Clin Nutr 71, 345–352. 27876809 10.1038/ejcn.2016.223 PMC6449850

[50] Cannon B and Nedergaard J (2004) Brown adipose tissue: function and physiological significance. Physiol Rev 84, 277–359. 14715917 10.1152/physrev.00015.2003

[51] Orava J , Nuutila P , Lidell ME , Oikonen V , Noponen T , Viljanen T , Scheinin M , Taittonen M , Niemi T , Enerbäck S *et al* (2011) Different metabolic responses of human brown adipose tissue to activation by cold and insulin. Cell Metab 14, 272–279. 21803297 10.1016/j.cmet.2011.06.012

[52] Bartelt A , Bruns OT , Reimer R , Hohenberg H , Ittrich H , Peldschus K , Kaul MG , Tromsdorf UI , Weller H , Waurisch C *et al* (2011) Brown adipose tissue activity controls triglyceride clearance. Nat Med 17, 200–205. 21258337 10.1038/nm.2297

[53] Chondronikola M , Volpi E , Børsheim E , Porter C , Annamalai P , Enerbäck S , Lidell ME , Saraf MK , Labbe SM , Hurren NM *et al* (2014) Brown adipose tissue improves whole‐body glucose homeostasis and insulin sensitivity in humans. Diabetes 63, 4089–4099. 25056438 10.2337/db14-0746 PMC4238005

[54] van Marken Lichtenbelt WD and Schrauwen P (2011) Implications of nonshivering thermogenesis for energy balance regulation in humans. Am J Physiol Regul Integr Comp Physiol 301, R285–R296. 21490370 10.1152/ajpregu.00652.2010

[55] Hauton D , Richards SB and Egginton S (2006) The role of the liver in lipid metabolism during cold acclimation in non‐hibernator rodents. Comp Biochem Physiol B Biochem Mol Biol 144, 372–381. 16730468 10.1016/j.cbpb.2006.03.013

[56] Shiota M , Tanaka T and Sugano T (1985) Effect of norepinephrine on gluconeogenesis in perfused livers of cold‐exposed rats. Am J Physiol 249 (3 Pt 1), E281–E286. 4037079 10.1152/ajpendo.1985.249.3.E281

[57] Penner PE and Himms‐Hagen J (1968) Gluconeogenesis in rats during cold acclimation. Can J Biochem 46, 1205–1213. 4301215 10.1139/o68-180

[58] Trayhurn P (1979) Fatty acid synthesis *in vivo* in brown adipose tissue, liver and white adipose tissue of the cold‐acclimated rat. FEBS Lett 104, 13–16. 477972 10.1016/0014-5793(79)81075-3

[59] Nakatsuka H , Shoji Y and Tsuda T (1983) Effects of cold exposure on gaseous metabolism and body composition in the rat. Comp Biochem Physiol A Comp Physiol 75, 21–25. 6133670 10.1016/0300-9629(83)90038-5

[60] Satapati S , Sunny NE , Kucejova B , Fu X , He TT , Méndez‐Lucas A , Shelton JM , Perales JC , Browning JD and Burgess SC (2012) Elevated TCA cycle function in the pathology of diet‐induced hepatic insulin resistance and fatty liver. J Lipid Res 53, 1080–1092. 22493093 10.1194/jlr.M023382 PMC3351815

[61] Vacanti NM , Divakaruni AS , Green CR , Parker SJ , Henry RR , Ciaraldi TP , Murphy AN and Metallo CM (2014) Regulation of substrate utilization by the mitochondrial pyruvate carrier. Mol Cell 56, 425–435. 25458843 10.1016/j.molcel.2014.09.024 PMC4267523

[62] Bricker DK , Taylor EB , Schell JC , Orsak T , Boutron A , Chen YC , Cox JE , Cardon CM , Van Vranken JG , Dephoure N *et al* (2012) A mitochondrial pyruvate carrier required for pyruvate uptake in yeast, *Drosophila*, and humans. Science 337, 96–100. 22628558 10.1126/science.1218099 PMC3690818

[63] Yang C , Ko B , Hensley CT , Jiang L , Wasti AT , Kim J , Sudderth J , Calvaruso MA , Lumata L , Mitsche M *et al* (2014) Glutamine oxidation maintains the TCA cycle and cell survival during impaired mitochondrial pyruvate transport. Mol Cell 56, 414–424. 25458842 10.1016/j.molcel.2014.09.025 PMC4268166

[64] Gray LR , Sultana MR , Rauckhorst AJ , Oonthonpan L , Tompkins SC , Sharma A , Fu X , Miao R , Pewa AD , Brown KS *et al* (2015) Hepatic mitochondrial pyruvate carrier 1 is required for efficient regulation of gluconeogenesis and whole‐body glucose homeostasis. Cell Metab 22, 669–681. 26344103 10.1016/j.cmet.2015.07.027 PMC4754674

[65] McCommis KS , Chen Z , Fu X , McDonald WG , Colca JR , Kletzien RF , Burgess SC and Finck BN (2015) Loss of mitochondrial pyruvate carrier 2 in the liver leads to defects in gluconeogenesis and compensation via pyruvate‐alanine cycling. Cell Metab 22, 682–694. 26344101 10.1016/j.cmet.2015.07.028 PMC4598280

[66] Bowman CE , Zhao L , Hartung T and Wolfgang MJ (2016) Requirement for the mitochondrial pyruvate carrier in mammalian development revealed by a hypomorphic allelic series. Mol Cell Biol 36, 2089–2104. 27215380 10.1128/MCB.00166-16 PMC4946427

[67] Cavaggioni A and Mucignat‐Caretta C (2000) Major urinary proteins, alpha(2U)‐globulins and aphrodisin. Biochim Biophys Acta 1482, 218–228. 11058763 10.1016/s0167-4838(00)00149-7

[68] Gómez‐Baena G , Armstrong SD , Phelan MM , Hurst JL and Beynon RJ (2014) The major urinary protein system in the rat. Biochem Soc Trans 42, 886–892. 25109974 10.1042/BST20140083

[69] Mucignat‐Caretta C , Caretta A and Cavaggioni A (1995) Acceleration of puberty onset in female mice by male urinary proteins. J Physiol 486 (Pt 2), 517–522. 7473215 10.1113/jphysiol.1995.sp020830 PMC1156539

[70] Chamero P , Marton TF , Logan DW , Flanagan K , Cruz JR , Saghatelian A , Cravatt BF and Stowers L (2007) Identification of protein pheromones that promote aggressive behaviour. Nature 450, 899–902. 18064011 10.1038/nature05997

[71] Hui X , Zhu W , Wang Y , Lam KSL , Zhang J , Wu D , Kraegen EW , Li Y and Xu A (2009) Major urinary protein‐1 increases energy expenditure and improves glucose intolerance through enhancing mitochondrial function in skeletal muscle of diabetic mice. J Biol Chem 284, 14050–14057. 19336396 10.1074/jbc.M109.001107 PMC2682853

[72] Charkoftaki G , Wang Y , McAndrews M , Bruford EA , Thompson DC , Vasiliou V and Nebert DW (2019) Update on the human and mouse lipocalin (LCN) gene family, including evidence the mouse Mup cluster is result of an “evolutionary bloom”. Hum Genomics 13, 11. 30782214 10.1186/s40246-019-0191-9 PMC6381713

[73] Zhang Y , Guo H , Deis JA , Mashek MG , Zhao M , Ariyakumar D , Armien AG , Bernlohr DA , Mashek DG and Chen X (2014) Lipocalin 2 regulates brown fat activation via a nonadrenergic activation mechanism. J Biol Chem 289, 22063–22077. 24917675 10.1074/jbc.M114.559104 PMC4139221

[74] Guo H , Foncea R , O'Byrne SM , Jiang H , Zhang Y , Deis JA , Blaner WS , Bernlohr DA and Chen X (2016) Lipocalin 2, a regulator of retinoid homeostasis and retinoid‐mediated thermogenic activation in adipose tissue. J Biol Chem 291, 11216–11229. 27008859 10.1074/jbc.M115.711556 PMC4900269

[75] Bonet ML , Oliver J , Picó C , Felipe F , Ribot J , Cinti S and Palou A (2000) Opposite effects of feeding a vitamin A‐deficient diet and retinoic acid treatment on brown adipose tissue uncoupling protein 1 (UCP1), UCP2 and leptin expression. J Endocrinol 166, 511–517. 10974645 10.1677/joe.0.1660511

[76] Sundaresan PR , Winters VG and Therriault DG (1967) Effect of low environmental temperature on the metabolism of vitamin A (retinol) in the rat. J Nutr 92, 474–478. 6058230 10.1093/jn/92.4.474

[77] Suski M , Olszanecki R , Madej J , Totoń‐Żurańska J , Niepsuj A , Jawień J , Bujak‐Giżycka B , Okoń K and Korbut R (2011) Proteomic analysis of changes in protein expression in liver mitochondria in apoE knockout mice. J Proteomics 74, 887–893. 21406262 10.1016/j.jprot.2011.03.003

[78] Jornayvaz FR and Shulman GI (2010) Regulation of mitochondrial biogenesis. Essays Biochem 47, 69–84. 20533901 10.1042/bse0470069 PMC3883043

