# Supplementary figures and images for: Comparative proteomic study of liver lipid droplets and mitochondria in mice housed at different temperatures
Source: FEBS Lett. 2019 Jul 12;593(16):2118–38. doi: 10.1002/1873-3468.13509 (PMC6771624; doi:10.1002/1873-3468.13509)

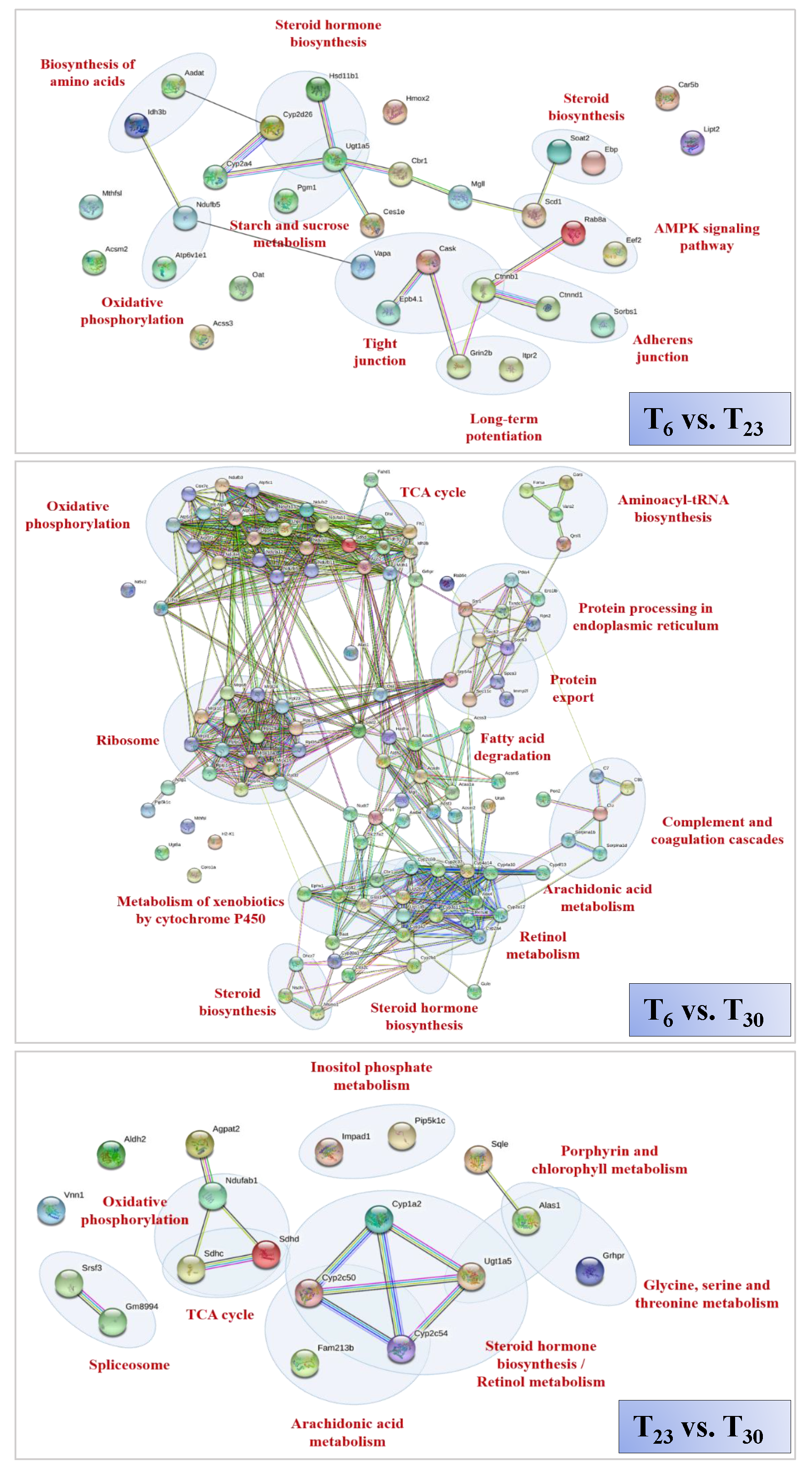

Supplement: Supplementary file 1 — Fig. S1. The association network of differential proteins in the liver mitochondria from mice living at different temperatures. [file FEB2-593-2118-s001.tif]

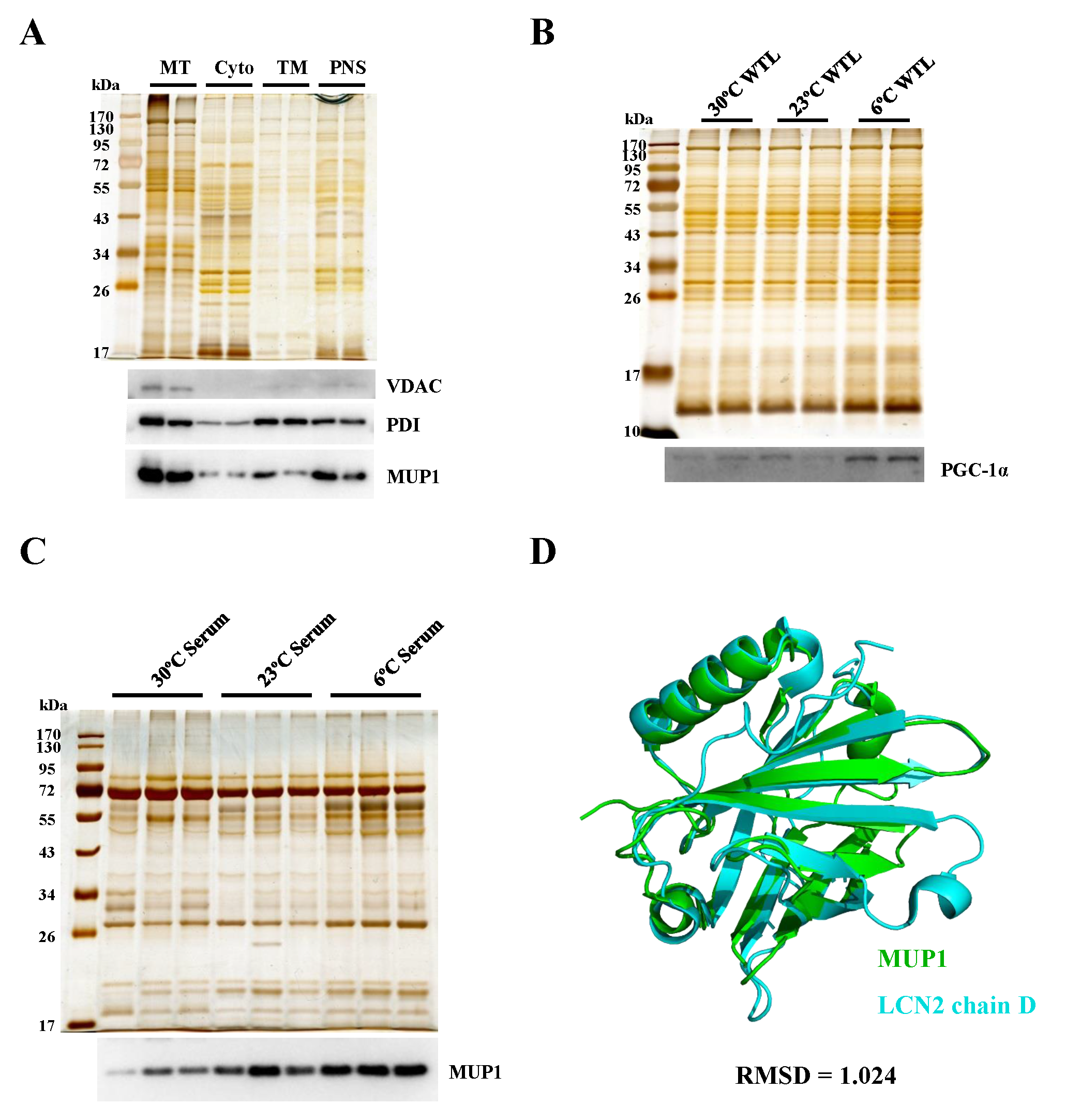

Supplement: Supplementary file 2 — Fig. S2. Subcellular localization of MUP1 and its possible function in mice living in a cold environment. [file FEB2-593-2118-s002.tif]
