# Supplementary material for: Comparative proteomic study of liver lipid droplets and mitochondria in mice housed at different temperatures
Source: FEBS Lett. 2019 Jul 12;593(16):2118–38. doi: 10.1002/1873-3468.13509 (PMC6771624; doi:10.1002/1873-3468.13509)
Supplement: Supplementary file 6 — Appendix S1. Supplemental information for mass spectrometry and Figs S1 and S2. [file FEB2-593-2118-s006.docx]

**Supplemental Information for**

**Comparative proteomic study on liver lipid droplets and mitochondria from mouse housed in different temperatures**

Qingfeng Liu^1^, Ziyun Zhou^2^, Pingsheng Liu^1,2,3^*, and Shuyan Zhang^2^*

**Appendix S1.**

**Materials and Methods**

**LC-MS/MS analysis**

Nano LC-MS/MS experiments were performed on a Q Exactive (Thermo Fisher Scientific) equipped with an Easy n-LC 1000 HPLC system (Thermo Fisher Scientific). The labeled peptides were loaded onto a 100 μm id×2 cm fused silica trap column packed in-house with reversed phase silica (Reprosil-Pur C18-AQ, 5 μm, Dr. Maisch GmbH) and then separated on a 75 μm id×20 cm C18 column packed with reversed phase silica (Reprosil-Pur C18-AQ, 3 μm, Dr. Maisch GmbH). The peptides bound on the column were eluted with a 78-min linear gradient. The solvent A consisted of 0.1% formic acid in water solution and the solvent B consisted of 0.1% formic acid in acetonitrile solution. The segmented gradient was 5–8% B, 8 min; 8–22% B, 50 min; 22–32% B, 12 min; 32-95% B, 1 min; 95% B, 7min at a flow rate of 300 nl/min.

The MS analysis was performed with Q Exactive mass spectrometer (Thermo Fisher Scientific). With the data-dependent acquisition mode, the MS data were acquired at a high resolution 70,000 (m/z 200) across the mass range of 300–1600 m/z. The target value was 3.00E+06 with a maximum injection time of 60 ms. The top 20 precursor ions were selected from each MS full scan with isolation width of 2 m/z for fragmentation in the HCD collision cell with normalized collision energy of 30%. Subsequently, MS/MS spectra were acquired at resolution setting of 17,500 at m/z 200. The target value was 5.00E+04 with a maximum injection time of 80 ms. The dynamic exclusion time was 40 s. For nano electrospray ion source setting, the spray voltage was 2.0 kV; no sheath gas flow; the heated capillary temperature was 320°C.

**Protein identification and quantification**

The raw data from Q Exactive were analyzed with Proteome Discovery version 2.2.0.388 using Sequest HT search engine for protein identification and Percolator for FDR (false discovery rate) analysis. The UniProt mouse protein database (updated on 10-2017) was used for searching the data. Searching parameters were set as follows: trypsin was selected as enzyme and two missed cleavages were allowed for searching; the mass tolerance of precursor was set as 10 ppm and the product ions tolerance was 0.02 Da; TMT 6plex (lysine and N-terminus of peptides) and cysteine carbamidomethylation were specified as fixed modifications; the methionine oxidation was chosen as variable modification. FDR analysis was performed using Percolator and FDR <1% was set for protein identification. The peptide confidence was set as high for peptide filter. Protein quantification was also performed on Proteome Discovery 2.2.0.388 using the ratio of the intensity of reporter ions from the MS/MS spectra. Only unique and razor peptides of proteins were selected for protein relative quantification. The co-isolation threshold was specified as 50% and average reporter S/N value should be above 10. The normalization to the protein median of each sample was used to correct experimental bias and the normalization mode was selected as total peptide amount.

**Supplemental Figure S1. The association network of differential proteins in the liver mitochondria from mouse living in different temperatures.**

The differentially expressed proteins in the T_6_ versus T_23_ group, T_6_ versus T_30_ group, and T_23_ versus T_30_ group were mapped to study the interaction by using STRING database (https://string-db.org/). Only those proteins enriched in the pathways with *P* value less than 0.05 were analyzed.

**Supplemental Figure S2. Subcellular localization of MUP1 and its possible function in mice living in cold environment.**

A. Subcellular localization of liver MUP1 in mice living under standard temperature was analyzed by biochemical method. Briefly, the proteins from isolated mitochondrion (MT), cytosol (Cyto), total membrane (TM), and post-nuclear supernatant (PNS) fractions were separated by SDS-PAGE and sliver stained (upper panel). Indicated antibodies to probe for marker proteins of different organelles were tested (lower panel); VDAC, (mitochondrion protein), PDI (ER protein). B. The expression of PGC-1α in liver of mice living in different temperatures was tested by Western Blotting. C. MUP1 in serum of mice living in different temperatures was tested by Western Blotting. D. The structures of MUP1 and lipocalin 2 were compared. Briefly, the structure data files of MUP1 and lipocalin 2 were downloaded from Protein Data Bank (http://www.rcsb.org/). Then their tertiary structures were superposed in molecular visualization system PyMOL (https://pymol.org/2/) and Root Mean Square Deviation (RMSD) value to indicate similarity was calculated.
